# Cross‐Sectional Survey on Mediastinal Lymph Node Dissection in Lung and Esophageal Cancer: A Project of the Anatomy of the Border Consensus Meeting at the 37th Annual Meeting of the Japan Society for Endoscopic Surgery

**DOI:** 10.1111/ases.70187

**Published:** 2025-11-23

**Authors:** Kentaro Miura, Koji Shindo, Yukihiro Terada, Toshiya Abe, Kenoki Ohuchida, Koichi Suda, Mingyon Mun, Kazutaka Obama, Masato Watanabe, Hisashi Iwata, Hisashi Shinohara, Ichiro Uyama, Hirokazu Noshiro, Norihiko Ikeda, Masafumi Nakamura, Yuko Kitagawa, Kimihiro Shimizu

**Affiliations:** ^1^ Division of General Thoracic Surgery, Department of Surgery Shinshu University School of Medicine Nagano Japan; ^2^ Department of Surgery and Oncology, Graduate School of Medical Sciences Kyushu University Fukuoka Japan; ^3^ Department of Surgery Fujita Health University Toyoake Japan; ^4^ Department of Thoracic Surgical Oncology Cancer Institute Hospital, Japanese Foundation for Cancer Research Tokyo Japan; ^5^ Department of Surgery, Graduate School of Medicine Kyoto University Kyoto Japan; ^6^ Department of Surgery Fukuoka University Chikushi Hospital Fukuoka Japan; ^7^ First Department of Surgery Gifu University School of Medicine Gifu Japan; ^8^ Department of Gastroenterological Surgery Hyogo Medical University Nishinomiya Japan; ^9^ Department of Advanced Robotic and Endoscopic Surgery Fujita Health University Toyoake Japan; ^10^ Department of Surgery, Faculty of Medicine Saga University Saga Japan; ^11^ Department of Surgery Tokyo Medical University Tokyo Japan; ^12^ Department of Surgery Keio University School of Medicine Tokyo Japan

**Keywords:** En bloc dissection, esophageal cancer surgery, Japan Society for Endoscopic Surgery, lung cancer surgery, mediastinal lymph node dissection

## Abstract

**Introduction:**

Although mediastinal lymph node dissection is performed in both lung and esophageal cancer surgeries, the underlying concepts and indications may differ between these fields. This study aimed to clarify these differences through a nationwide questionnaire survey initiated by the 37th Annual Meeting of the Japan Society for Endoscopic Surgery.

**Methods:**

A joint task force from the lung and esophageal surgery divisions developed a questionnaire focusing on four key areas: (i) lymph node dissection around the left recurrent nerve, (ii) subcarinal lymph node dissection, (iii) pulmonary ligament lymph node dissection, and (iv) en bloc lymph node dissection. The survey was distributed to certified core institutions across Japan.

**Results:**

The response rates were 50.4% for lung cancer institutions and 57.0% for esophageal cancer institutions. In the esophageal division, dissection of the aforementioned lymph nodes was routinely performed in most core institutions. In contrast, practices in the lung division varied widely, particularly depending on tumor location. The concept of “sampling” was rarely recognized in esophageal surgery but was partially accepted in lung surgery. Furthermore, there was no uniform definition of “en bloc dissection” across either field.

**Conclusion:**

This cross‐sectional survey revealed notable conceptual differences between lung and esophageal cancer divisions regarding mediastinal lymph node dissection, despite targeting the same anatomical regions. Additionally, significant variability was observed even within the lung division. These findings indicate a lack of standardized consensus in Japan and highlight the need for ongoing cross‐disciplinary dialog and consensus building.

## 1 Introduction

To perform safe and reliable surgery, a thorough understanding of precise anatomy is essential for all surgeons. Many anatomical structures are relevant to multiple surgical disciplines—for example, the relationship between the lungs and esophagus in thoracic anatomy, or between the lungs and liver in relation to the diaphragm. However, the approaches or conceptual frameworks applied to these anatomical regions may differ among specialties, as the target diseases involving those structures vary. To date, there have been no published discussions or reports that examine such cross‐disciplinary perspectives.

One example of cross‐disciplinary anatomy is mediastinal lymph node dissection, which is commonly performed in both lung and esophageal cancer surgeries. Although the same regional lymph nodes may be dissected, the purposes and conceptual significance of the procedure can differ between the two fields. Sharing this knowledge could contribute to the development of improved surgical strategies and may help reduce operative time by omitting unnecessary steps. However, due to the lack of comprehensive discussions or surveys on the subject, such differences remain unclear.

In lung cancer, various strategies exist for assessing lymph node metastasis, including lymph node sampling, lobe‐specific (selective) dissection, and systematic lymph node dissection. Some studies have reported that lobe‐specific or systematic dissection improves progression outcomes [1, 2, 3, 4, 5, 6, 7]. In contrast, other studies have concluded that lymph node dissection does not significantly influence prognosis [8, 9, 10]. Thus, the necessity of lymph node dissection in lung cancer remains a subject of ongoing debate.

In contrast, according to the Japanese guidelines [11, 12], surgical resection remains the standard treatment for esophageal cancer, although chemoradiotherapy is also accepted for early‐stage disease and as neoadjuvant therapy in advanced cases. Because esophageal cancer has a high propensity for lymph node metastasis, lymph node dissection is essential for achieving radical resection. While lymph node regions were previously categorized as upper, middle, and lower mediastinum, the 12th edition of the Japanese Classification of Esophageal Cancer defines lymph node stations more precisely by anatomical location [13]. Standardized surgical techniques for these regions have been established in several institutions [14, 15, 16].

To explore these differences in anatomical understanding and surgical concepts across disciplines, the Consensus Meeting of Anatomy on the Border (AoB Consensus Meeting) was organized as a special symposium at the 37th Annual Meeting of the Japan Society for Endoscopic Surgery (Fukuoka, Japan, 2024). This report focuses on one of the sessions, titled “Mediastinal Lymph Node Dissection,” which addresses an anatomical region common to both lung and esophageal cancers. Using a questionnaire survey, we investigated current practices and conceptual differences in mediastinal lymph node dissection between surgeries for stage I lung cancer and esophageal cancer.

## 2 Patients and Methods

### 2.1 Establishment of Steering Committee, Expert Committee, and Research Committee

To examine and clarify anatomical interpretations across disciplines through the *Anatomy on the Border (AoB) Consensus Meeting* at the 37th Annual Meeting of the Japan Society for Endoscopic Surgery, a Steering Committee was established in 2023 (Figure S1).

Subsequently, the Steering Committee selected members of the Expert Committee (EC), consisting of specialists in the fields of thoracic, esophageal, hepato‐biliary, urological, and hernia surgery. Members of the Research Committee (RC), responsible for conducting the research, were then appointed from each specialty. Finally, six interdisciplinary subgroups were formed: esophageal‐lung, hepatobiliary‐lung‐urinary, diaphragm, pediatric‐adult biliary duct, inguinal, and median arcuate ligament groups.

### 2.2 Clinical Questions and Questionnaire

The joint esophageal‐lung group formulated four clinical questions (CQ1–CQ4). The CQ3 group was a collaborative effort between the Division of General Thoracic Surgery, Department of Surgery, Shinshu University School of Medicine (EC: KS; RC: KM), the Department of Surgery and Oncology, Graduate School of Medical Sciences, Kyushu University, and the Department of Surgery, Faculty of Medicine, Saga University (EC: HN; RC: KS). The main theme of CQ3 was “Concepts of Lymph Node Dissection: Logic Based on Lymphatic Flow and Therapeutic Efficacy,” with three core clinical questions (CQ3‐1, CQ3‐2, and CQ3‐3) developed:

- CQ3‐1: Are there any differences in concepts or dissection targets around the left recurrent nerve lymph nodes between thoracic and esophageal surgery?
- CQ3‐2: Are there any differences in concepts or dissection targets of subcarinal lymph nodes between thoracic and esophageal surgery?
- CQ3‐3: Are there any differences in concepts or dissection targets of pulmonary ligament lymph nodes between thoracic and esophageal surgery?

Based on the CQ themes, we developed a questionnaire comprising comparable items for both surgical specialties wherever possible. The goal was to identify commonalities and differences in conceptual understanding, procedural indications, and surgical recognition between lung and esophageal cancer surgeries. The questionnaire focused on four key areas:

- Lymph node dissection around the left recurrent nerve.
- Subcarinal lymph node dissection.
- Pulmonary ligament lymph node dissection, and
- En bloc dissection techniques.

In the thoracic surgery group, the questionnaire included 24 items and was distributed to all certified Japanese training facilities in general thoracic surgery, accompanied by a cover letter explaining the study objectives. The survey targeted patients with clinical Stage I lung cancer undergoing anatomical lung resection (excluding wedge resection). All questionnaire items are listed in Supplemental Table S1.

In contrast, the esophageal surgery questionnaire, consisting of 14 items, was distributed to certified institutions specializing in esophageal surgery (Supplemental Table S2).

### 2.3 Literature Search

A literature search was conducted using PubMed. The keywords used included: *lung cancer*, *esophageal cancer*, *lymph node dissection*, *left recurrent*, *subcarinal*, and *pulmonary ligament*. The complete search formula is provided in Supplemental Table S3.

Only original English‐language articles and review papers were included; case reports were excluded.

### 2.4 Statements, Consensus, and Reporting

Joint statements were drafted based on both the questionnaire results and the literature review (Supplemental Table S4). Voting on each statement was conducted using the Delphi method by all EC and RC members of the esophageal–lung fusion group.

The final results were presented at the 37th Annual Meeting of the Japan Society for Endoscopic Surgery (Special Symposium, Fukuoka, Japan, 2024) for discussion and validation.

## 3 Results

### 3.1 Overview of Responses

**FIGURE 1:**
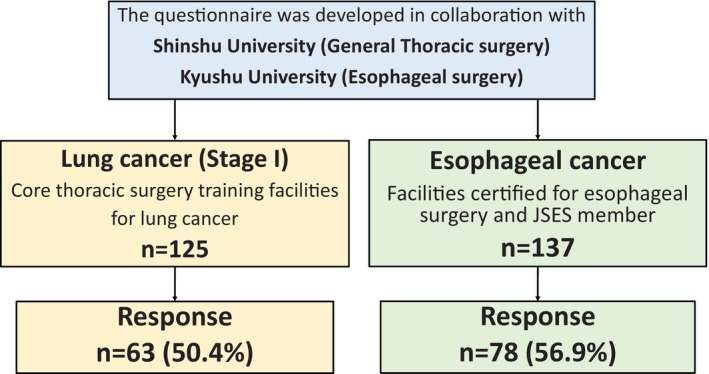
Flowchart of the questionnaire survey. The questionnaire was jointly developed by the Esophageal Surgery Group at Kyushu University and the General Thoracic Surgery Group at Shinshu University. The response rates were 57.0% (78 of 137 institutions) for esophageal surgery and 50.4% (63 of 125 institutions) for thoracic surgery.

A flow chart of the questionnaire distribution and response process is shown in Figure 1. In the lung division, 125 institutions were targeted, and responses were received from 63 institutions (50.4%). In the esophageal division, 137 institutions were targeted, and 79 institutions responded (57.7%). The results of the questionnaires from both divisions are presented below.

- **Lymph Node Dissection Around the Left Recurrent Laryngeal Nerve**.

**(Lung cancer: #4 L and #5; Esophageal cancer: #106recL)**.

**(Lung cancer: Questions 1–5; Esophageal cancer: Questions 1–2)**.

**(I) Indications for Lymph Node Dissection**.

**Lung Cancer (Questions 1–4)**.

**FIGURE 2:**
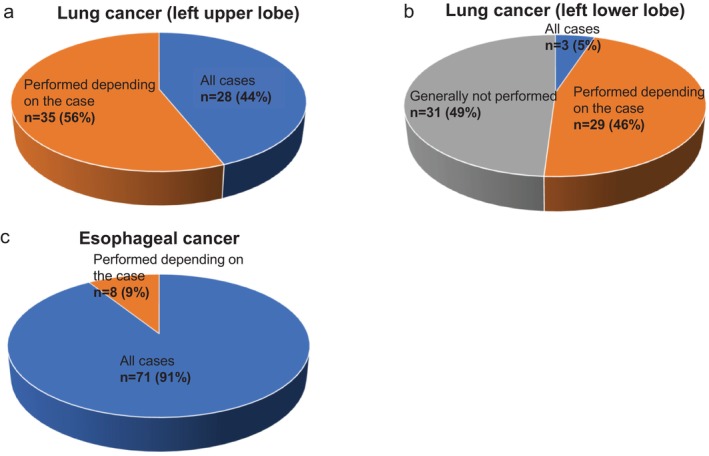
Indications for lymph node dissection around the recurrent laryngeal nerve (lung cancer: #4 L and #5; esophageal cancer: #106recL). (a) Lung cancer (left upper lobe), (b) Lung cancer (left lower lobe), and (c) Esophageal cancer.

The results are shown in Figure 2. In surgeries for the left upper lobe, 28 of 63 institutions (44%) reported performing dissection around the left recurrent laryngeal nerve lymph nodes in all cases, while 36 institutions (56%) indicated that the decision depends on the specific case. No institution reported that dissection is “Generally not performed” (Figure 2a).

In contrast, for left lower lobe surgeries, only 3 institutions (5%) reported performing the dissection in all cases. Meanwhile, 29 institutions (46%) responded “performed depending on the case,” and 31 institutions (49%) reported “generally not performed” (Figure 2b).

Institutions that answered “generally not performed” were asked about the criteria used for decision‐making. The responses are summarized in Supplemental Figure S2.

- For left upper lobe surgeries, the most common factor was “tumor size or malignancy” (*n* = 34/36, 94%), followed by:
  − “age or preoperative complications” (*n* = 30/36, 83%).
  − “SUV‐max value on PET‐CT” (*n* = 18/36, 50%).
  − “preoperative or intraoperative lymphadenopathy” (*n* = 16/36, 44%).
  − “intraoperative rapid diagnosis of hilar lymph node” (*n* = 15/36, 42%).
  − “tumor location” (*n* = 11/36, 31%) (Supplemental Figure S2a).
- For left lower lobe surgeries, responses were similar:
  − “tumor size or malignancy” (*n* = 26/29, 90%).
  − “age or preoperative complications” (*n* = 20/29, 69%).
  − “preoperative or intraoperative lymphadenopathy” (*n* = 16/29, 55%).
  − “SUV‐max value on PET‐CT” (*n* = 15/29, 52%).
  − “intraoperative rapid diagnosis of hilar lymph node” (*n* = 15/29, 52%).
  − “tumor location” (*n* = 11/29, 38%) (Supplemental Figure S2b).

**Esophageal Cancer (Question 1)**.

As shown in Figure 2c, 71 of 78 institutions (91%) reported that dissection around the left recurrent nerve lymph nodes is performed in all cases. Of the remaining 7 institutions:

- 4 (50%) responded that the decision depends on the primary tumor region, even in the absence of enlarged nodes.
- 3 (38%) responded that the decision depends on both the tumor site and lymph node size.
- 1 (13%) cited the clinical stage as the determining factor (Supplemental Figure S3).

**(II) Concepts of Lymph Node Dissection**.

**Lung Cancer (Question 5)**.

**FIGURE 3:**
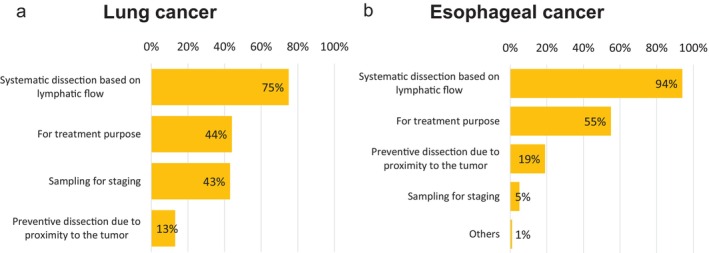
Conceptual approaches to lymph node dissection around the recurrent laryngeal nerve. (a) Lung cancer, and (b) Esophageal cancer.

The results are shown in Figure 3a.

When asked about the conceptual basis for left recurrent laryngeal nerve lymph node dissection:

- 47 of 63 institutions (75%) selected “systematic dissection based on lymphatic flow”.
- 28 institutions (44%) selected “for treatment purposes”.
- 27 institutions (43%) selected “sampling for staging”.
- 8 institutions (13%) selected “preventive dissection due to proximity to the tumor”.

**Esophageal Cancer (Question 2)**.

The results are shown in Figure 3b.

- 73 of 78 institutions (94%) responded “systematic dissection based on lymphatic flow”.
- 42 (55%) responded “for treatment purposes”.
- 15 (19%) selected “preventive dissection due to proximity to the tumor”.
- 4 (5%) selected “sampling for staging”.
- 1 (1%) selected “others”.

B **Subcarinal Lymph Node Dissection**.

**(Lung cancer: #7; Esophageal cancer: #107 and #109)**.

**(Lung cancer: Questions 6–14; Esophageal cancer: Questions 3–7)**.

**(I) Indications for Lymph Node Dissection**.

**Lung Cancer (Questions 6–10)**.

**FIGURE 4:**
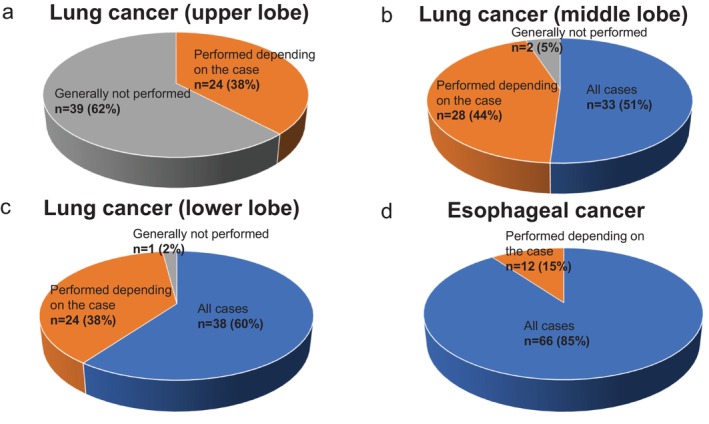
Indications for subcarinal lymph node dissection (lung cancer: #7; esophageal cancer: #107 and #109). (a) Lung cancer (upper lobe), (b) Lung cancer (middle lobe), (c) Lung cancer (lower lobe), and (d) Esophageal cancer.

The results are shown in Figure 4.

- **Upper lobe**: No institution reported performing subcarinal lymph node dissection in all cases. Instead, 24 of 63 institutions (38%) answered “performed depending on the case,” while 39 institutions (62%) responded “generally not performed” (Figure 4a).
- **Middle lobe**: 33 of 63 institutions (51%) reported performing dissection in all cases, 28 institutions (44%) responded “ performed depending on the case,” and 2 institutions (5%) responded “generally not performed” (Figure 4b).
- **Lower lobe**: 38 of 63 institutions (60%) reported performing dissection in all cases, 24 institutions (38%) answered “ performed depending on the case,” and 1 institution (2%) responded “generally not performed” (Figure 4c).

For institutions answering “ performed depending on the case,” the decision‐making criteria are shown in Supplemental Figure S4. The most frequently cited reasons included:

- “tumor size or malignancy” (*n* = 23/24, 96%).
- “age or preoperative complications” (*n* = 21/24, 88%).
- “SUV‐max value on PET‐CT” (*n* = 14/24, 58%).
- “preoperative or intraoperative lymphadenopathy” (*n* = 14/24, 58%).
- “tumor location” (*n* = 10/24, 42%).
- “intraoperative rapid diagnosis of hilar lymph node” (*n* = 9/24, 38%).

All 63 institutions (100%) answered that the concept of subcarinal lymph node dissection is consistent between the right and left sides (Supplemental Figure S5).

**Esophageal Cancer (Question 3)**.

As shown in Figure 4d, 66 of 78 institutions (85%) reported performing subcarinal lymph node dissection in all cases. Of the remaining 12 institutions:

- 7 (58%) answered that the indication depends on the primary tumor region.
- 3 (25%) responded that it depends on the primary site and lymph node size.
- 2 (17%) based the decision on clinical stage (Supplemental Figure S6).

**(II) Concepts and Expectations Regarding Prognosis**.

**Lung Cancer (Questions 11–12)**.

**FIGURE 5:**
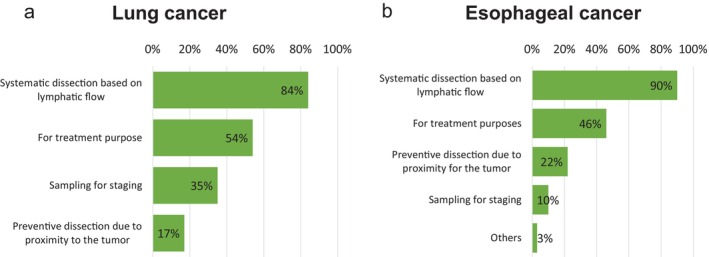
Concepts of subcarinal lymph node dissection. (a) Lung cancer, and (b) Esophageal cancer.

The results are shown in Figure 5a.

Regarding the conceptual approach to subcarinal lymph node dissection:

- 53 of 63 institutions (84%) selected “systematic dissection based on lymphatic flow”.
- 34 (54%) selected “for treatment purposes”.
- 22 (35%) selected “sampling for staging”.
- 11 (17%) selected “preventive dissection due to proximity to the tumor”.

In terms of expectations regarding prognosis:

- 51 institutions (81%) answered “some expectations”.
- 4 institutions (6%) answered “very high expectations”.
- 8 institutions (13%) answered “no expectations” (Supplemental Figure S7a).

**Esophageal Cancer (Questions 4–5)**.

The results are shown in Figure 5b.

- 70 of 78 institutions (90%) selected “systematic dissection based on lymphatic flow”.
- 36 (46%) selected “for treatment purposes”.
- 17 (22%) selected “preventive dissection due to proximity to the tumor”.
- 8 (10%) selected “sampling for staging”.
- 2 (3%) selected “others”

In terms of expectations:

- 67 institutions (86%) answered “some expectations”.
- 6 institutions (8%) answered “very high expectations”.
- 5 institutions (6%) answered “no expectations” (Supplemental Figure S7b).

**(III) Lymphatic Flow**.

**Lung Cancer (Questions 13–14)**.

Responses and discussion of lymphatic flow around subcarinal lymph nodes are described in Supplemental Text S1.

**Esophageal Cancer (Questions 6–7)**.

Findings are presented in Supplemental Text S2.

C **Pulmonary Ligament Lymph Node Dissection**.

**(Lung: #9; Esophagus: #112)**.

**(Lung cancer: Questions 15–22; Esophageal cancer: Questions 8–12)**.

**(I) Indications for Lymph Node Dissection**.

**Lung Cancer (Questions 15–18)**.

**FIGURE 6:**
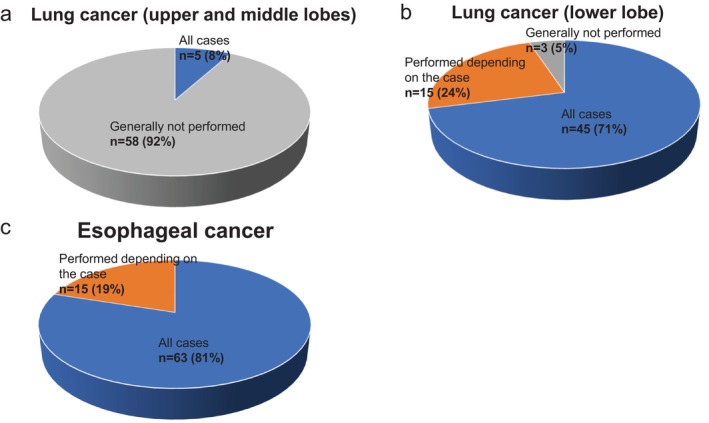
Indications for pulmonary ligament lymph node dissection (lung cancer: #9; esophageal cancer: #112). (a) Lung cancer (upper and middle lobes), (b) Lung cancer (lower lobe), and (c) Esophageal cancer.

As shown in Figure 6a, for upper and middle lobes:

- 58 of 63 institutions (92%) reported that pulmonary ligament lymph node dissection is “generally not performed”.
- 5 institutions (8%) answered “performed in all cases”.

For lower lobe cases (Figure 6b):

- 45 institutions (71%) reported dissection is performed in all cases.
- 15 (24%) answered “performed depending on the case”.
- 3 (5%) answered “generally not performed”.

Decision criteria from the “case‐dependent” group are shown in Supplemental Figure S10. Common reasons included:

- “age or preoperative complications” (*n* = 12/15, 80%).
- “tumor size or malignancy” (*n* = 12/15, 80%).
- “preoperative or intraoperative lymphadenopathy” (*n* = 10/15, 67%).
- “SUV‐max value on PET‐CT” (*n* = 7/15, 47%).
- “tumor location” (*n* = 6/15, 40%).
- “intraoperative rapid diagnosis of hilar lymph node” (*n* = 4/15, 27%).

All institutions (63/63, 100%) responded that the concepts are the same between the right and left sides (Supplemental Figure S11).

**Esophageal Cancer (Question 8)**.

As shown in Figure 6c, 63 of 78 institutions (81%) reported performing pulmonary ligament lymph node dissection in all cases. Of the remaining 15:

- 11 (73%) based the decision on the primary tumor region.
- 3 (20%) based it on both tumor site and lymph node size.
- 1 (7%) based it on clinical stage (Supplemental Figure S12).

**(II) Concepts and Expectations Regarding Prognosis**.

**Lung Cancer (Questions 19–20)**.

**FIGURE 7:**
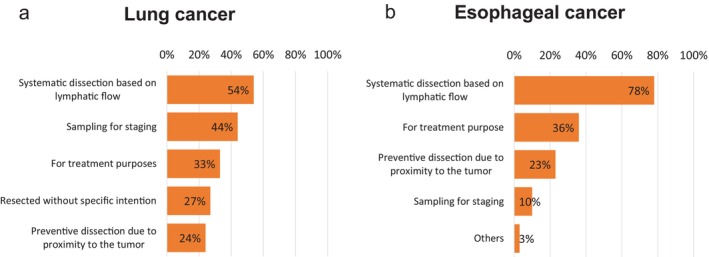
Concepts of pulmonary ligament lymph node dissection. (a) Lung cancer, and (b) Esophageal cancer.

Results are shown in Figure 7a.

In terms of dissection concept:

- 34 institutions (54%) answered “systematic dissection based on lymphatic flow”.
- 28 (44%) answered “sampling for staging”.
- 21 (33%) answered “for treatment purposes”.
- 17 (27%) answered “resected without specific intention”.
- 15 (24%) answered “preventive dissection due to proximity to the tumor”.

Expectations regarding prognosis:

- 32 institutions (51%) answered “some expectations”.
- 3 (5%) answered “very high expectations”.
- 28 (44%) answered “no expectations” (Supplemental Figure S13a).

**Esophageal Cancer (Question 9)**.

The results are shown in Figure 7b.

- 61 of 78 institutions (78%) answered “systematic dissection based on lymphatic flow”.
- 28 (36%) answered “for treatment purposes”.
- 18 (23%) answered “preventive dissection due to proximity to the tumor”.
- 8 (10%) answered “sampling for staging”.
- 2 (3%) answered “others”.

Expectations:

- 58 institutions (74%) answered “some expectations”.
- 4 (5%) answered “very high expectations”.
- 16 (21%) answered “no expectations” (Supplemental Figure S13b).

**(III) Lymphatic Flow**.

**Lung Cancer (Questions 21–22)**.

Details are described in Supplemental Text S3.

**Esophageal Cancer (Questions 11–12)**.

Described in Supplemental Text S4.

D **En Bloc Lymph Node Dissection**.

**(Lung cancer: Questions 23–24; Esophageal cancer: Questions 13–14)**.

We asked participating institutions about the concept and implementation of en bloc lymph node dissection.

- **Lung Cancer (Questions 23–24)**.
  ○ Details are described in Supplemental Text S5.
- **Esophageal Cancer (Questions 13–14)**.
  ○ Details are described in Supplemental Text S6.

E **Summary of Commonalities and Differences Between Lung and Esophageal Divisions**.

Table 1 summarizes the commonalities and differences in mediastinal lymph node dissection between lung and esophageal surgeries.

**TABLE 1:** The commonalities and differences between lung and esophageal fields.

Region	The commonalities	The differences
Around Recurrent Nerve Lymph Node Dissection	Over 70% of respondents in both fields reported performing “systematic dissection with attention to lymphatic flow.”	In the esophageal field, over 90% reported performing dissection in all cases, whereas in the lung field, practice varied by lobe. The concept of “sampling” was rarely recognized in the esophageal field.
Subcarinal lymph node dissection	Both fields showed a similar conceptual approach, favoring systematic dissection based on lymphatic flow. Approximately 80% in both groups responded that the prognostic impact is “some expectation.” Recognition of lymphatic flow direction (upstream/downstream) was varied.	In the esophageal field, more than 80% reported routine dissection, while in the lung field, it varied by lobe. The concept of “sampling” was scarcely acknowledged in esophageal cancer.
Pulmonary ligament lymph node dissection	Recognition of upstream and downstream lymphatic flow was inconsistent in both groups.	In lung cancer, dissection was performed in over 70% of lower lobe cases but was rare for upper/middle lobes. In contrast, 80% of esophageal cases underwent dissection regardless of tumor location. In the concepts, 44% answered “sampling” in the lungs, while less than 10% in the esophagus. In the lung group, 44% recognized “sampling” as a concept, compared to less than 10% in the esophageal group. Regarding prognostic impact, 44% of lung surgeons answered “no impact,” while 74% of esophageal surgeons answered “some expectation.”
En bloc lymph node dissection	About half of respondents considered en bloc dissection “clinically significant but technically difficult.” Around 50% defined en bloc dissection as “regional lymph node dissection without division.”	In the lung field, 29% considered en bloc dissection “meaningless,” whereas only 5% in the esophageal field shared this view.

In brief:

- Dissections around the left recurrent nerve, subcarinal, and pulmonary ligament lymph nodes are routinely performed in most esophageal surgery institutions, whereas indications vary significantly across institutions in lung surgery, particularly depending on tumor location.
- The concept of “sampling” is relatively uncommon in esophageal surgery, while it is more accepted in lung surgery.
- In terms of expected postoperative prognosis, responses regarding subcarinal lymph node dissection were relatively consistent across both fields. However, for pulmonary ligament lymph node dissection, esophageal surgeons tended to express greater expectations compared to thoracic surgeons.
- Regarding the lymphatic flow around subcarinal and pulmonary ligament lymph nodes, no unified or standardized view was observed in either field.

Regarding en bloc lymph node dissection, approximately half of the institutions in both lung and esophageal groups considered it to be “clinically meaningful but technically difficult,” typically defining it as regional lymph node dissection without dividing the surrounding tissue.

In contrast, 29% of lung surgery institutions considered en bloc dissection “meaningless,” while only 5% of esophageal surgery institutions shared that opinion.

F **Literature Search**.

A literature review was conducted using PubMed, targeting three anatomical regions:

- Left recurrent laryngeal nerve lymph node dissection: 49 articles.
- Subcarinal lymph node dissection: 138 articles.
- Pulmonary ligament lymph node dissection: 48 articles.

From these, we selected:

- 17 studies relevant to lung cancer.
- 16 studies relevant to esophageal cancer.

Only original English‐language research articles and review papers were included; case reports were excluded from this review.

### 3.2 Statement

The final statement was formulated based on the analysis of both the lung and esophageal division questionnaires, as well as a review of relevant literature (Supplemental Table S4).

A summary of the statement is as follows:

First, there are substantial differences in concepts and indications regarding mediastinal lymph node dissection between institutions specializing in lung and esophageal cancers.

Second, differences in concepts and indications also exist within each surgical specialty.

Third, the establishment of a shared consensus on these concepts and indications may help reduce institutional variation and contribute to the standardization of surgical quality.

Detailed explanations for each clinical question are also provided.

Members of the Expert Committee (EC) and Research Committee (RC) from both lung and esophageal divisions voted on the statement. It was unanimously approved with a 100% affirmative vote (21 out of 21), using the Delphi method.

The results and statement were presented and formally endorsed during the 37th Annual Meeting of the Japan Society for Endoscopic Surgery (Special Symposium, Fukuoka, Japan, 2024).

## 4 Discussion

This cross‐sectional study using a questionnaire survey revealed several important findings: (i) The concepts and recognition of mediastinal lymph node dissection differ in multiple aspects between lung and esophageal cancers, despite targeting the same anatomical regions. (ii) Furthermore, even within each individual specialty, institutional variations in conceptual understanding were observed among core facilities.

This was the first attempt to conduct such a cross‐sectional, multidisciplinary survey. The process of sharing these findings across specialties led to new insights and the formulation of a consensus statement.

### 4.1 Commonalities and Differences Between Lung and Esophageal Divisions

When comparing lung and esophageal surgery divisions, both similarities and differences were identified in lymph node dissection practices, specifically regarding the left recurrent laryngeal nerve, subcarinal, and pulmonary ligament nodes.

In the esophageal division, systematic mediastinal lymph node dissection is generally performed regardless of tumor location or clinical stage. In contrast, in the lung division, indications for dissection often depend on tumor location, malignancy, and related factors.

Interestingly, institutions from both specialties reported having some expectations regarding the prognostic impact of mediastinal lymph node dissection. However, there were considerable differences in the perception of the clinical significance and technical feasibility of en bloc dissection, as well as in the understanding of lymphatic flow pathways.

Ultimately, no unified consensus exists within or between the two specialties regarding these procedures.

The following discussions provide further detail on the findings for mediastinal lymph node dissection in each field, as described in Supplemental Texts S7 (lung cancer) and S8 (esophageal cancer).

### 4.2 Outlook for the Future

As discussed above, the value of mediastinal lymph node dissection in lung cancer—beyond its role in pathological staging—remains unclear and controversial. With the rapid development of perioperative chemotherapy regimens (e.g., Felip, IMpower010 [17]; Herbst, ADAURA [18]), accurate pathological staging has become increasingly beneficial for guiding treatment strategies. The results of JCOG1413, the first large‐scale prospective trial evaluating whether the extent of nodal dissection affects overall survival in early‐stage non‐small cell lung cancer, is currently under analysis [19]. This trial may help resolve the long‐standing debate regarding the necessity of extensive lymph node dissection in lung cancer.

In contrast, the prognostic value of lymph node dissection in esophageal cancer has been well documented [20, 21]. In particular, a prospective study on lymph node dissection at the tracheal bifurcation demonstrated a significant survival difference depending on whether the node was dissected [22]. Mediastinal lymph node dissection remains an essential component of esophageal cancer surgery.

Reaching a unified consensus on mediastinal lymph node dissection across both lung and esophageal cancer may be difficult due to differences in tumor biology, malignancy potential, and mechanisms of progression. However, developing a shared understanding within each field could bring clinical benefits and promote collaborative insights. Greater recognition of these differences may ultimately help generate new evidence and refine surgical concepts.

### 4.3 Limitations

Several limitations must be acknowledged. First, the survey targeted only Japanese institutions, with response rates of 50.4% for thoracic surgery and 57.7% for esophageal surgery. Second, there was limited opportunity for direct discussion of the results between both specialties, although they were presented at the 37th Annual Meeting of the Japan Society for Endoscopic Surgery (Special Symposium, 2024, Fukuoka). Given these limitations, the findings should not be interpreted as definitive evidence. However, this initiative fostered mutual understanding of conceptual differences and clinical approaches between the two fields. We believe that this study and the resulting consensus statement will contribute to the future development of surgical strategies in both lung and esophageal cancer.

## 5 Conclusions

This cross‐sectional study clarified the current status of mediastinal lymph node dissection in both lung and esophageal cancer surgeries. Despite targeting the same anatomical regions, significant differences in conceptual approaches were observed between the two fields. While the necessity and effectiveness of these procedures remain subjects for future investigation, establishing a shared understanding of their roles—even in the absence of high‐level evidence—may help reduce inter‐institutional variation and contribute to the standardization of surgical care across disciplines.

## Conflicts of Interest

Kenoki Ohuchida, Kazutaka Obama, Mingyon Mun, and Koichi Suda are the Editorial Board members of ASES Journal and the co‐authors of this article. To minimize bias, they were excluded from all editorial decision‐making related to the acceptance of this article for publication. Koichi Suda does not have conflicts of interest or financial relationships to disclose regard to the present study. However, K.S. received funding from Sysmex Co. in relation to the Collaborative Laboratory for Research and Development in Advanced Surgical. Intelligence, Fujita Health University. K.S. also received research expenses from Sysmex, Co., unrelated to the submitted work. Kazutaka Oabama has received honoraria from Sysmex Corporation, Medicaroid Corporation, Olympus Corporation, Covidien Japan Inc., Intuitive Surgical G.K., and Ethicon Inc. Ichiro uyama has received honoraria from Asahi Intecc Co., Intuitive Surgical G.K., Medtronic Corporation and Medicaroid Corporation. I. U. is affiliated with the research and development in advanced surgical technology endowed chair at Medicaroid Inc. Hirokazu Noshiro has received honoraria from Medicaroid Corporation and Intuitive Surgical Inc. Hisashi Iwata has received honoraria from Intuitive Surgical G.K. Norihiko Ikeda has received research grants to the department from AstraZeneca, Chugai. Pharmaceutical, Pfizer, Taiho Pharmaceutical, MSD, Boehringer Ingelheim, Eli Lilly, Ono. Pharmaceutical, Teijin, Nihon Mediphysics, Fujifilm, and Johnson and Johnson, and honoraria from AstraZeneca, Chugai Pharmaceutical, Pfizer, Taiho Pharmaceutical, MSD, Boehringer Ingelheim, Eli Lilly, Ono Pharmaceutical, Teijin, Nihon Mediphysics, Bristol‐Myers, Olympus, Medtronic, and Johnson and Johnson. Masafumi Nakamura has received research funding from Olympus Corporation, Taiho. Pharmaceutical Co. Ltd., Covidien Japan Inc., Chugai Pharmaceutical Co. Ltd., Eli Lilly. Japan K.K., and Otsuka Pharmaceutical Co. Ltd., and honoraria from Intuitive Surgical. G.K., Johnson and Johnson K.K., Yakult Honsha Co. Ltd., Taiho Pharmaceutical Co. Ltd., Daiichi Sankyo Co. Ltd., Otsuka Pharmaceutical Co. Ltd., Novartis Pharma K.K., Olympus Corporation, Covidien Japan Inc., and Servier Japan Co. Ltd. Yuko Kitagawa has received honoraria from Sysmex Corporation, Medicaroid Corporation, Olympus Corporation, Stryker Japan K.K., Intuitive Surgical G.K., and Ethicon Inc, research funding from Medicaroid Corporation. The others do not have conflicts of interest or financial relationships to disclose with regard to the present study.

## Supporting information


**Table S1:Supplemental** Lung Division Questionnaire.

**Table S2:Supplemental** Esophageal Division Questionnaire.

**Table S4:Supplemental** Joint Statement and Explanatory Notes of the Survey.

**Data S1:** Supporting Information.

## Data Availability

The data that support the findings of this study are available from the corresponding author upon reasonable request.
