# Supplementary material for: Cross‐Sectional Survey on Mediastinal Lymph Node Dissection in Lung and Esophageal Cancer: A Project of the Anatomy of the Border Consensus Meeting at the 37th Annual Meeting of the Japan Society for Endoscopic Surgery
Source: Asian J Endosc Surg. 2025 Nov 23;18(1):e70187. doi: 10.1111/ases.70187 (PMC12640797; doi:10.1111/ases.70187)
Supplement: Supplementary file 1 — Table S1:Supplemental Lung Division Questionnaire. [file ASES-18-e70187-s004.docx]

**Supplemental Table S1** – Lung Division Questionnaire

English-translated version for international submission

Section 1: Left Recurrent Laryngeal Nerve Lymph Node Dissection (#4L and #5)

Q1. Do you perform #4L and #5 dissection in cases of left upper lobe lung cancer?

 a. Performed in all cases

 b. Generally not performed

 c. Performed depending on the case

Q2. If you selected “c” in Question 1, which of the following criteria do you consider? (Multiple answers allowed)

 a. Age or preoperative comorbidities

 b. Tumor location

 c. Tumor size or malignancy (including GGN component)

 d. SUV-max value on PET-CT

 e. Preoperative or intraoperative lymphadenopathy

 f. Intraoperative rapid diagnosis of hilar lymph nodes

 g. Other (please specify): ___________

Q3. Do you perform #4L and #5 dissection in cases of left lower lobe lung cancer?

 a. Performed in all cases

 b. Generally not performed

 c. Performed depending on the case

Q4. If you selected “c” in Question 3, which of the following criteria do you consider? (Multiple answers allowed)

 [Same as Q2]

Q5. What is your primary concept or purpose for #4L and #5 lymph node dissection?

 a. Systematic dissection based on lymphatic flow

 b. Sampling for staging

 c. Therapeutic intent

 d. Preventive dissection due to proximity to the tumor

 e. Other (please specify): ___________

Section 2: Subcarinal Lymph Node Dissection (#7)

Q6. Do you perform subcarinal lymph node (#7) dissection in lower lobe lung cancer?

 a. Performed in all cases

 b. Generally not performed

 c. Performed depending on the case

Q7. If you selected “c” in Question 6, which of the following criteria do you consider? (Multiple answers allowed)

 [Same as Q2]

Q8. Is there a difference in subcarinal lymph node dissection between the left and right sides?

 a. Yes

 b. No

Q9. Do you perform subcarinal lymph node (#7) dissection in upper lobe lung cancer?

 a. Performed in all cases

 b. Generally not performed

 c. Performed depending on the case

Q10. Do you perform subcarinal lymph node (#7) dissection in middle lobe lung cancer?

 a. Performed in all cases

 b. Generally not performed

 c. Performed depending on the case

Q11. What is your primary concept or purpose for subcarinal lymph node dissection?

 [Same as Q5]

Q12. How much impact do you expect subcarinal lymph node dissection to have on prognosis?

 a. High impact

 b. Moderate expectations

 c. No impact

Q13. Where is the upstream lymphatic flow to the subcarinal lymph node?

 a. Along the esophagus

 b. Anterior to the trachea

 c. From the hilum

 d. Along both main bronchi

 e. Other (please specify): ___________

Q14. Where is the downstream lymphatic flow from the subcarinal lymph node?

 [Same as Q13]

Section 3: Pulmonary Ligament Lymph Node Dissection (#9)

Q15. Do you perform pulmonary ligament lymph node dissection in lower lobe lung cancer?

 a. Performed in all cases

 b. Generally not performed

 c. Performed depending on the case

Q16. If you selected “c” in Question 15, which of the following criteria do you consider? (Multiple answers allowed)

 [Same as Q2]

Q17. Is there a difference in pulmonary ligament lymph node dissection between the left and right sides?

 a. No, the same

 b. Yes, different

Q18. Do you perform pulmonary ligament lymph node dissection in upper or middle lobe lung cancer?

 a. Yes

 b. Generally not performed

Q19. What is your primary concept or purpose for pulmonary ligament lymph node dissection?

 [Same as Q5]

Q20. How much impact do you expect pulmonary ligament lymph node dissection to have on prognosis?

 [Same as Q12]

Q21. Where is the upstream lymphatic flow to the pulmonary ligament lymph node?

 a. Esophageal hiatus

 b. Along the esophagus

 c. From the hilum

 d. Pericardial fat tissue

 e. Other (please specify): ___________

Q22. Where is the downstream lymphatic flow from the pulmonary ligament lymph node?

 [Same as Q21]

Section 4: En Bloc Lymph Node Dissection

Q23. In your opinion, is en bloc lymph node dissection necessary?

 a. Required

 b. Ideally meaningful, but technically difficult

 c. Ideally meaningful, but technically impossible

 d. Not necessary

 e. Other (please specify): ___________

Q24. How do you define “en bloc” lymph node dissection?

 a. Partial contiguous dissection with the lung

 b. Regional lymph node dissection without division

 c. Dissection of all lymph nodes without tissue separation

 d. Technically impossible

 e. Other (please specify): ___________
