# Supplementary material for: Cross‐Sectional Survey on Mediastinal Lymph Node Dissection in Lung and Esophageal Cancer: A Project of the Anatomy of the Border Consensus Meeting at the 37th Annual Meeting of the Japan Society for Endoscopic Surgery
Source: Asian J Endosc Surg. 2025 Nov 23;18(1):e70187. doi: 10.1111/ases.70187 (PMC12640797; doi:10.1111/ases.70187)
Supplement: Supplementary file 2 — Table S2:Supplemental Esophageal Division Questionnaire. [file ASES-18-e70187-s002.docx]

**Supplemental Table S2** – Esophageal Division Questionnaire

Mediastinal Lymph Node Dissection in Esophageal Cancer

Section 1: Left Recurrent Laryngeal Nerve Lymph Node Dissection (#106recL)

Q1. Do you perform lymph node dissection around the left recurrent laryngeal nerve (#106recL) during radical esophagectomy?

 a. Performed in all cases

 b. Depends on the primary tumor location, even if no lymph node enlargement is present

 c. Depends on both tumor location and lymph node size, even if no lymph node enlargement is present

 d. Performed according to clinical stage

 e. Only if there is an enlarged lymph node in this region

 f. Other (please specify): ___________

Q2. What is your concept for performing lymph node dissection around the left recurrent laryngeal nerve? (Multiple answers allowed)

 a. Systematic dissection with attention to lymphatic flow (including prophylactic dissection in cN0)

 b. Sampling for staging

 c. Therapeutic dissection (including post-neoadjuvant chemotherapy)

 d. Prophylactic dissection due to proximity to the tumor

 e. Other (please specify): ___________

Section 2: Subcarinal Lymph Node Dissection (#107 and #109)

Q3. Do you perform lymph node dissection around the tracheal bifurcation (#107, #109)?

 a. Performed in all cases

 b. Depends on the primary tumor location, even if no lymph node enlargement is present

 c. Depends on both tumor location and lymph node size, even if no lymph node enlargement is present

 d. Performed according to clinical stage

 e. Only if there is an enlarged lymph node in this region

 f. Other (please specify): ___________

Q4. What is your concept for lymph node dissection around the tracheal bifurcation? (Multiple answers allowed)

 [Same answer options as Q2]

Q5. How much impact do you expect subcarinal lymph node dissection to have on prognosis?

 a. High impact

 b. Moderate expectations

 c. No impact (primarily for staging)

 d. Other (please specify): ___________

Q6. What is the upstream lymphatic flow to the subcarinal lymph node? (Multiple answers allowed)

 a. Along the esophagus

 b. Cephalad direction (anterior to the trachea)

 c. From the pulmonary hilum

 d. Along the left and right main bronchi

 e. Other (please specify): ___________

Q7. What is the downstream lymphatic flow from the subcarinal lymph node? (Multiple answers allowed)

 a. Cephalad direction (anterior to the trachea)

 b. Along the left and right main bronchi

 c. To the thoracic duct

 d. Along the esophagus

 e. To the pulmonary hilum

 f. Other (please specify): ___________

Section 3: Pulmonary Ligament Lymph Node Dissection (#112)

Q8. Do you perform lymph node dissection around the inferior pulmonary ligament (#112)?

 a. Performed in all cases

 b. Depends on the primary tumor location, even if no lymph node enlargement is present

 c. Depends on both tumor location and lymph node size, even if no lymph node enlargement is present

 d. Performed according to clinical stage

 e. Only if there is an enlarged lymph node in this region

 f. Other (please specify): ___________

Q9. What is your concept for lymph node dissection around the pulmonary ligament? (Multiple answers allowed)

 [Same answer options as Q2]

Q10. How much impact do you expect pulmonary ligament lymph node dissection to have on prognosis?

 a. High impact

 b. Moderate expectations

 c. No impact (primarily for staging)

Q11. What is the upstream lymphatic flow to the pulmonary ligament lymph node?

 a. From the esophageal hiatus (abdominal region)

 b. Along the esophagus

 c. Along the lung

 d. From the pericardial fat tissue

 e. Other (please specify): ___________

Q12. What is the downstream lymphatic flow from the pulmonary ligament lymph node?

 [Same options as Q11]

Section 4: En Bloc Lymph Node Dissection

Q13. Please select the option that best describes your opinion on en bloc dissection in radical esophagectomy:

 a. Required

 b. Ideally meaningful but technically difficult

 c. Ideally meaningful but technically impossible

 d. Not necessary

 e. Other (please specify): ___________

Q14. Which of the following best describes your definition of "en bloc" lymph node dissection?

 a. When lymphatic tissue is partially continuous with the esophagus

 b. When lymphatic tissue is dissected without dividing the region

 c. Not possible because it disrupts the drainage pathway to the cervical lymph nodes

 d. When mediastinal lymphatic tissue is removed in a single block, even if cervical nodes are dissected separately

 e. Other (please specify): ___________
