# Supplementary material for: Cross‐Sectional Survey on Mediastinal Lymph Node Dissection in Lung and Esophageal Cancer: A Project of the Anatomy of the Border Consensus Meeting at the 37th Annual Meeting of the Japan Society for Endoscopic Surgery
Source: Asian J Endosc Surg. 2025 Nov 23;18(1):e70187. doi: 10.1111/ases.70187 (PMC12640797; doi:10.1111/ases.70187)
Supplement: Supplementary file 3 — Table S4:Supplemental Joint Statement and Explanatory Notes of the Survey. [file ASES-18-e70187-s003.docx]

**Supplemental Table S4. Joint Statement and Explanatory Notes of the Survey**

**Clinical Questions (CQ3)**

- **CQ3-1:** Are there differences in the concepts and indications for lymph node dissection around the left recurrent laryngeal nerve between the lung and esophageal fields?
- **CQ3-2:** Are there differences in the concepts and indications for subcarinal lymph node dissection between the lung and esophageal fields?
- **CQ3-3:** Are there differences in the concepts and indications for pulmonary ligament lymph node dissection between the lung and esophageal fields?

**Joint Statement: Conceptual Basis of Lymph Node Dissection (Lymphatic Flow and Therapeutic Significance)**

- There are notable differences among institutions in both the lung and esophageal fields regarding the significance of lymph node dissection in the regions surrounding the left recurrent nerve, subcarinal, and pulmonary ligament.
- In lung cancer, the extent of mediastinal lymph node dissection is generally determined based on tumor location. Conversely, in esophageal cancer—particularly thoracic esophageal lesions—systematic dissection is widely applied regardless of tumor location. Systematic dissection with attention to lymphatic flow is broadly accepted as the ideal approach in esophageal surgery. However, in thoracic surgery, systematic dissection is not universally considered necessary, and there is substantial variation in how its value is perceived.
- The concept and feasibility of en bloc lymph node dissection remain controversial. There is limited evidence supporting its efficacy, and opinions differ significantly, particularly in the lung field.
- While further studies are needed to clarify the therapeutic role of these procedures, developing a shared understanding—even in the absence of robust evidence—may help reduce inter-institutional variation and promote the standardization of surgical practice across both specialties.

**Explanatory Notes**

**Introduction**

Dissection of lymph nodes near the left recurrent laryngeal nerve, subcarinal, and pulmonary ligament is considered potentially beneficial for tumor control and prognostic improvement in both lung and esophageal cancers. However, the indications and surgical strategies differ between fields. Additionally, concepts such as *en bloc dissection* and the anatomical understanding of lymphatic flow show variation among institutions and surgeons.

This survey was conducted among:

- 79 certified esophageal surgery centers, and
- 63 core thoracic surgery training facilities in Japan.
  The lung cancer portion was limited to clinical Stage I, due to variability in treatment approaches for Stage II/III.

**1. Left Recurrent Laryngeal Nerve Lymph Node Dissection**

- In esophageal surgery, dissection around the left recurrent nerve (#106recL) is standard practice for thoracic esophageal cancer, performed regardless of tumor location or stage. It is recognized for improving local control and reducing metastasis, supported by multiple studies [1,2].
- In contrast, for clinical Stage I lung cancer, dissection of corresponding nodes (#4L, #5) is performed selectively. In upper lobe cases, 35 institutions indicated "case-by-case" dissection, while 28 chose "all cases." For lower lobe, only 3 institutions answered "all cases," with 31 "case-by-case" and 29 "not performed."
  This suggests significant variability in practice, with decisions often based on tumor location, patient condition, imaging, and tumor characteristics (e.g., GGN component) [3–5].

**2. Subcarinal Lymph Node Dissection**

- In esophageal surgery, subcarinal lymph node dissection (#107, #109) is widely recognized as contributing to metastasis prevention and improved prognosis [6–8]. According to the survey, 70 out of 78 institutions adopt systematic dissection with lymphatic flow awareness.
- In lung surgery, practice is more varied.
   – **Upper lobe cancer**: No institutions perform dissection routinely; 24 "case-by-case", 39 "not performed."
   – **Middle lobe**: 33 "all cases", 28 "case-by-case."
   – **Lower lobe**: 38 "all cases", 24 "case-by-case."

Studies suggest that dissection may be omitted in upper lobectomy, explaining the variability [9–11]. While lymphatic pathways are considered in decision-making, tumor location strongly influences dissection strategy.

**3. Pulmonary Ligament Lymph Node Dissection**

- For esophageal cancer (#112), 63 institutions reported routine dissection, while 16 stated it depends on tumor location or size. Literature supports its role in reducing metastasis and improving outcomes [12].
- In lung cancer (#9), 45 institutions routinely dissect for lower lobe tumors, but only 5 do so for upper/middle lobes. Previous studies suggest that the value of dissection varies based on tumor location [13–15]. Thus, unlike esophageal surgery, dissection in lung surgery is more selective.

**4. En Bloc Lymph Node Dissection**

- En bloc dissection, defined as removing lymphatic tissue with the tumor in one block, is viewed as ideal by many esophageal surgeons, but technical feasibility limits its application.

 – **Esophageal field**: 27 institutions answered "ideally meaningful, but technically difficult"; 18 "not meaningful."
 – **Lung field**: 14 "essential", 18 "not meaningful", 27 "technically difficult", 3 "technically impossible."

- No clinical studies currently demonstrate a survival benefit for en bloc dissection in either field, and no standard definition exists.

**5. Lymphatic Flow Recognition**

- Understanding lymphatic pathways is critical for rational dissection planning [16,17]. However, survey results showed substantial variability in perceptions between institutions.

**Subcarinal Lymph Node (Upstream)**

- Esophageal: Anterior to trachea (n=39), hilum (n=33), along esophagus (n=30), both main bronchi (n=29)
- Lung: Anterior to trachea (n=30), hilum (n=30), both main bronchi (n=28), esophagus (n=13)

**Subcarinal Lymph Node (Downstream)**

- Esophageal: Anterior to trachea (n=36), both main bronchi (n=33)
- Lung: Both main bronchi (n=33), anterior to trachea (n=25), esophagus (n=19), hilum (n=18)

**Pulmonary Ligament Lymph Node (Upstream)**

- Lung: Along lung (n=36), esophagus (n=30), hilum (#10/#11) (n=26), esophagus (#8) (n=21), esophageal hiatus (n=8), pericardial fat (n=5)

**Pulmonary Ligament Lymph Node (Downstream)**

- Esophageal: Esophageal hiatus (n=27), lung (n=21), esophagus (n=18), pericardial fat (n=10)
- Lung: Esophageal hiatus (n=24), esophagus (#8) (n=18), hilum (#10/#11) (n=10), pericardial fat (n=8)

These results confirm that there is no consensus across or within specialties regarding lymphatic flow recognition.

**Conclusion and Future Direction**

Although lymph node dissection in the left recurrent nerve, subcarinal, and pulmonary ligament areas is acknowledged as clinically meaningful in both lung and esophageal cancers, the criteria and approaches vary significantly.

In esophageal surgery, systematic dissection is standard for thoracic esophageal lesions. In contrast, in lung surgery, dissection is more individualized, primarily based on tumor location.

Moreover, there are marked inter-institutional differences in views on en bloc dissection and lymphatic flow pathways.

As such, this study underscores the absence of a shared cross-disciplinary consensus.
Reducing these conceptual differences may help diminish disparities in clinical practice and foster the standardization of care across institutions and specialties.

**References**

1. Akiyama H, Tsurumaru M, Udagawa H, Kajiyama Y. Radical lymph node dissection for cancer of the thoracic esophagus. Ann Surg. 1994;220(3):364–372.
2. Shimada H, Okumura M, Matsubara H, et al. Japan Esophageal Society classification of lymph node metastasis from esophageal cancer based on therapeutic value. J Esophagus. 2021;18(1):1–9.
3. Darling GE, Allen MS, Decker PA, et al. Randomized trial of mediastinal lymph node sampling versus complete lymphadenectomy during pulmonary resection in the patients with N0 or N1 (less than hilar) non-small cell carcinoma: results of the ACOSOG Z0030 trial. J Thorac Cardiovasc Surg. 2011;141(3):662–670.
4. Okada M, Nishio W, Sakamoto T, et al. Selective mediastinal lymphadenectomy for clinical stage I non-small cell lung cancer. Ann Thorac Surg. 2006;81(3):1028–1032.
5. Kobayashi Y, Sakai T, Saito Y, et al. Prognostic impact of subcarinal lymph node dissection for right upper lobe NSCLC. J Thorac Dis. 2021;13(4):2153–2162.
6. Tachimori Y, Nagai Y, Kanamori N, et al. Pattern of lymph node metastases in thoracic esophageal carcinoma. Cancer. 1998;82(9):1756–1763.
7. Chino O, Watanabe M, Ishizuka N, et al. Is the extent of lymphadenectomy associated with outcome in esophageal cancer? Dis Esophagus. 2006;19(4):254–259.
8. Mine S, Sano T, Fujisaki J, et al. Lymphatic spread of esophageal squamous cell carcinoma. Ann Thorac Cardiovasc Surg. 2011;17(5):457–465.
9. Suzuki K, Watanabe SI, Wakabayashi M, et al. A non-randomized confirmatory trial of sublobar resection for ground-glass opacity–dominant peripheral lung cancer: JCOG0804/WJOG4507L. J Clin Oncol. 2020;38(26):2933–2942.
10. Kanzaki R, Kanzaki M, Yamamoto M, et al. Lymphatic pathway and surgical consideration for segmentectomy. Gen Thorac Cardiovasc Surg. 2021;69(5):824–830.
11. Chen FF, Yan YJ, Wang YB, et al. Pulmonary ligament lymph node dissection in NSCLC surgery: significance and clinical outcomes. Thorac Cancer. 2022;13(15):2122–2129.
12. Tamura S, Yamasaki M, Ishihara R, et al. Lymph node metastasis in early-stage esophageal squamous cell carcinoma: significance and predictive factors. Esophagus. 2018;15(1):34–41.
13. Wang J, Wu N, Lv C, et al. Effect of different surgical approaches to pulmonary ligament lymph node dissection in lung cancer. BMC Cancer. 2022;22(1):458.
14. Fujisawa T, Yamada Y, Okumura M, et al. Surgical perspectives on lymphadenectomy in lung cancer. Gen Thorac Cardiovasc Surg. 2020;68(12):1233–1241.
15. Aokage K, Yoshida J, Ishii G, et al. Subcarinal lymph node dissection and prognosis in patients with right upper lobe NSCLC. J Thorac Oncol. 2010;5(1):71–75.
16. Riquet M, Le Pimpec-Barthes F, Dujon A, et al. Anatomical basis and strategy of lymph node dissection in lung cancer. Thorac Surg Clin. 2012;22(2):197–206.
17. Tsuboi M, Ishikawa Y, Imai K, et al. Lymphatic flow and clinical relevance in thoracic surgery. J Thorac Dis. 2020;12(6):3098–3105.
