# Supplementary material for: Cross‐Sectional Survey on Mediastinal Lymph Node Dissection in Lung and Esophageal Cancer: A Project of the Anatomy of the Border Consensus Meeting at the 37th Annual Meeting of the Japan Society for Endoscopic Surgery
Source: Asian J Endosc Surg. 2025 Nov 23;18(1):e70187. doi: 10.1111/ases.70187 (PMC12640797; doi:10.1111/ases.70187)
Supplement: Supplementary file 4 — Data S1: Supporting Information. [file ASES-18-e70187-s001.docx]

**Title:** Cross-sectional Survey on Mediastinal Lymph Node Dissection in Lung and Esophageal Cancer: A Project of the Anatomy of the Border Consensus Meeting at the 37th Annual Meeting of the Japan Society for Endoscopic Surgery

**Authors:** Kentaro Miura*^1^,MD, Koji Shindo*^2^, MD, Yukihiro Terada^1^, MD, Toshiya Abe^2^, MD, Kenoki Ohuchida^2^, MD, Koichi Suda^3^, MD, Mingyon Mun^4^, MD, Kazutaka Obama^5^, MD, Masato Watanabe^6^, MD, Hisashi Iwata^7^, MD, Hisashi Shinohara^8^, MD, Ichiro Uyama^9^, MD, Hirokazu Noshiro^10^, MD, Norihiko Ikeda^11^, MD, Masafumi Nakamura^2^, MD, Yuko Kitagawa^12^, MD, and Kimihiro Shimizu^1^ , MD, PhD

*K.M. and K.S. contributed equally to this work.

**Affiliation:**

^1^Division of General Thoracic Surgery, Department of Surgery, Shinshu University School of Medicine, 3-1-1, Asahi, Matsumoto, Nagano, 390-8621, Japan

^2^Department of Surgery and Oncology, Graduate School of Medical Sciences, Kyushu University, 3-1-1 Maidashi, Higashi-ku, Fukuoka, Fukuoka, 812-8582, Japan

^3^Department of Surgery, Fujita Health University, 1-98 Dengakugakubo, Kutsukake, Toyoake, Aichi, 470-1192, Japan.

^4^Department of Thoracic Surgical Oncology, Cancer Institute Hospital, Japanese Foundation for Cancer Research, 3-8-31 Ariake, Koto-ku, Tokyo 135-8550, Japan.

^5^Department of Surgery, Graduate School of Medicine, Kyoto University, 54 Shogoin‐ Kawara‐cho, Sakyo‐ku, Kyoto 606‐8507, Japan.

^6^Department of Surgery, Fukuoka University Chikushi Hospital, 1-1-1 Zokumyoin, Chikushino, Fukuoka 818-8502, Japan.

^7^First Department of Surgery, Gifu University School of Medicine, 40 Tsukasa, Gifu 500-8705, Japan.

^8^Department of Gastroenterological Surgery, Hyogo Medical University, 1-1 Mukogawa-cho, Nishinomiya, Hyogo, 663-8501, Japan.

^9^Department of Advanced Robotic and Endoscopic Surgery, Fujita Health University, Toyoake, Aichi, 470-1192, Japan.

^10^Department of Surgery, Faculty of Medicine, Saga University, 5-1-1 Nabeshima, Saga, 849-8501, Japan.

^11^Department of Surgery, Tokyo Medical University, 6-7-1 Nishishinjuku, Shinjuku-ku, Tokyo, 160-0023, Japan.

^12^Department of Surgery Keio University School of Medicine, 35 Shinanomachi, Shinju-ku, Tokyo 160-8582, Japan.

**Correspondence to:**

Kimihiro Shimizu

Division of General Thoracic Surgery, Department of Surgery, Shinshu University School of Medicine
3-1-1 Asahi, Matsumoto, 390-8621, Japan

Telephone: +81-263-37-3576

E-mail: [kmshimizu@gmail.com](mailto:kmshimizu@gmail.com)

**CONTENTS**

**Supplementary Texts**

- **Supplementary Text S1.** Lymphatic flow around subcarinal lymph nodes in lung cancer
- **Supplementary Text S2.** Lymphatic flow around subcarinal lymph nodes in esophageal cancer
- **Supplementary Text S3.** Lymphatic flow around pulmonary ligament lymph nodes in lung cancer
- **Supplementary Text S4.** Lymphatic flow around pulmonary ligament lymph nodes in esophageal cancer
- **Supplementary Text S5.** Results of en bloc lymph node dissection in lung cancer
- **Supplementary Text S6.** Results of en bloc lymph node dissection in esophageal cancer
- **Supplementary Text S7.** Discussion on lymph node dissection and en bloc dissection in lung cancer
- **Supplementary Text S8.** Discussion on lymph node dissection and en bloc dissection in esophageal cancer

**Supplementary Tables**

- **Supplementary Table S1.** Questionnaire for the lung division (separate file)
- **Supplementary Table S2.** Questionnaire for the esophageal division (separate file)
- **Supplementary Table S3.** Literature search strategy used for statement development
- **Supplementary Table S4.** Joint statement and explanatory notes of the survey (separate file)

**Supplementary Figures**

- **Supplementary Figure S1.** Summary of study methods
- **Supplementary Figure S2.** Criteria for left recurrent nerve lymph node dissection in lung cancer: (a) left upper lobe, (b) left lower lobe
- **Supplementary Figure S3.** Criteria for left recurrent nerve lymph node dissection in esophageal cancer
- **Supplementary Figure S4.** Criteria for subcarinal lymph node dissection in lung cancer (lower lobe)
- **Supplementary Figure S5.** Responses to “Are subcarinal lymph node dissections different between left and right sides?” (lung cancer)
- **Supplementary Figure S6.** Criteria for subcarinal lymph node dissection in esophageal cancer
- **Supplementary Figure S7.** Expectations for subcarinal lymph node dissection: (a) lung cancer, (b) esophageal cancer
- **Supplementary Figure S8.** Upstream lymphatic flow of subcarinal lymph nodes: (a) lung cancer, (b) esophageal cancer
- **Supplementary Figure S9.** Downstream lymphatic flow of subcarinal lymph nodes: (a) lung cancer, (b) esophageal cancer
- **Supplementary Figure S10.** Criteria for pulmonary ligament lymph node dissection in lung cancer
- **Supplementary Figure S11.** Responses to “Are subcarinal lymph node dissections different between left and right sides?” (lung cancer)
- **Supplementary Figure S12.** Criteria for pulmonary ligament lymph node dissection in esophageal cancer
- **Supplementary Figure S13.** Expectations for pulmonary ligament lymph node dissection: (a) lung cancer, (b) esophageal cancer
- **Supplementary Figure S14.** Upstream lymphatic flow of pulmonary ligament lymph nodes: (a) lung cancer, (b) esophageal cancer
- **Supplementary Figure S15.** Downstream lymphatic flow of pulmonary ligament lymph nodes: (a) lung cancer, (b) esophageal cancer
- **Supplementary Figure S16.** Responses to “Is en bloc lymph node dissection required?”: (a) lung cancer, (b) esophageal cancer
- **Supplementary Figure S17.** Definitions of “en bloc” lymph node dissection: (a) lung cancer, (b) esophageal cancer

**Supplementary Text S1.** **Lymphatic Flow Around Subcarinal Lymph Nodes in Lung Cancer**

**(Related to Questions 13 and 14)**

Among 63 responding institutions, 30 (48%) identified the upstream lymphatic flow of subcarinal lymph nodes as either "anterior to the trachea" or "hilum." This was followed by "along both main bronchi" (28 institutions, 44%) and "along the esophagus" (13 institutions, 21%) (**Supplementary Figure S8a**).

For downstream flow, 33 institutions (52%) selected "along both main bronchi," followed by "anterior to the trachea" (25 institutions, 40%), "along the esophagus" (19 institutions, 30%), and "hilum" (18 institutions, 29%) (**Supplementary Figure S9a**).

**Supplementary Text S2. Lymphatic Flow Around Subcarinal Lymph Nodes in Esophageal Cancer**

**(Related to Questions 6 and 7 – Multiple choices allowed)**

Of the 78 responding institutions, 38 (49%) identified the upstream lymphatic flow of subcarinal lymph nodes as "cephalad (anterior to the trachea)," followed by "hilum" (32 institutions, 41%), "along the esophagus" (29 institutions, 37%), and "along the main bronchi" (28 institutions, 36%) (**Supplementary Figure S8b**).

For downstream flow, 35 institutions (45%) selected "cephalad (anterior to the trachea)," followed by "along the main bronchi" (33 institutions, 42%), "hilum" (15 institutions, 19%), "along the esophagus" (9 institutions, 12%), and "thoracic duct" (6 institutions, 8%) (**Supplementary Figure S9b**).

**Supplementary Text S3. Lymphatic Flow Around Pulmonary Ligament Lymph Nodes in Lung Cancer**

**(Related to Questions 21 and 22)**

When asked about upstream flow to the pulmonary ligament lymph nodes, 26 out of 63 institutions (41%) selected "hilum," followed by "along the esophagus" (21 institutions, 33%), "esophageal hiatus" (8 institutions, 13%), and "pericardial fat tissue" (5 institutions, 8%) (**Supplementary Figure S14a**).

For downstream flow, 24 institutions (38%) selected "esophageal hiatus," followed by "along the esophagus" (18 institutions, 29%), "hilum" (10 institutions, 16%), and "pericardial fat tissue" (8 institutions, 13%) (**Supplementary Figure S15a**).

**Supplementary Text S4. Lymphatic Flow Around Pulmonary Ligament Lymph Nodes in Esophageal Cancer**

**(Related to Questions 11 and 12)**

Among 78 institutions, 36 (46%) identified the upstream lymphatic flow of pulmonary ligament lymph nodes as "along the lungs," followed by "along the esophagus" (29 institutions, 37%), "esophageal hiatus" (6 institutions, 8%), and "pericardial fat tissue" (5 institutions, 6%) (**Supplementary Figure S14b**).

Regarding downstream flow, 26 institutions (33%) selected "esophageal hiatus," followed by "along the lungs" (21 institutions, 27%), "along the esophagus" (18 institutions, 23%), and "pericardial fat tissue" (10 institutions, 13%) (**Supplementary Figure S15b**).

**Supplementary Text S5. En Bloc Lymph Node Dissection in Lung Cancer**

**(Related to Questions 23 and 24)**

In response to whether en bloc lymph node dissection is required, 14 of 63 institutions (22%) answered "required." The majority (27 institutions, 43%) selected "ideally meaningful, but technically difficult," while 3 (5%) responded "ideally meaningful, but technically impossible." Additionally, 18 institutions (29%) considered it "meaningless" (**Supplementary Figure S16a**).

When asked how en bloc dissection is defined, the most frequent answer was "regional lymph node dissection without division" (31 institutions, 49%), followed by "dissection of all lymph nodes without division" (23 institutions, 37%), "partial contiguous dissection with lung" (5 institutions, 8%), and "impossible" (2 institutions, 3%) (**Supplementary Figure S17a**).

**Supplementary Text S6. En Bloc Lymph Node Dissection in Esophageal Cancer**

**(Related to Questions 13 and 14)**

Among the 78 respondents, 15 (19%) answered that en bloc dissection is "required." The majority selected either "ideally meaningful, but technically difficult" (44 institutions, 56%) or "technically impossible" (11 institutions, 14%). Only 4 (5%) considered the procedure "meaningless." Some institutions added that en bloc dissection may be feasible depending on the location or condition of the lymph nodes, while others pointed out the need for a clearer definition (**Supplementary Figure S16b**).

Regarding definitions, 38 of 78 institutions (49%) defined en bloc dissection as "dissection of lymphatic tissue without dividing it." Two additional categories were selected equally (each by 14 institutions, 18%):

(1) "En bloc dissection is not possible because it disrupts the drainage pathway to the cervical lymph nodes" and

(2) "Dissection of mediastinal lymphatic tissue in one block, even if cervical lymph nodes are addressed separately" (**Supplementary Figure S17b**).

These findings underscore the need to clearly define en bloc dissection, especially considering that esophagectomy with lymphadenectomy involves three anatomical fields—cervical, mediastinal, and abdominal—potentially disrupting the continuity of lymphatic drainage.

**Supplemental text S7.** Discussion of lymph node dissection and en bloc dissection in lung cancer.

**1. Left recurrent nerve lymph node dissection**

For upper lobe lung cancer, all institutions reported performing dissection of lymph nodes around the left recurrent laryngeal nerve either routinely or on a case-by-case basis. However, for lower lobe cancer, approximately half of the institutions responded that dissection was “basically not performed.”

The necessity of dissection in this region remains controversial, with no consensus in the literature. Deng et al. retrospectively analyzed the nodal metastasis pattern of 590 patients with clinical stage I NSCLC and found that no tumors ≤2 cm in the lower lobe metastasized to upper mediastinal lymph nodes. They concluded that upper mediastinal node dissection may be unnecessary for such small tumors [1]. Yang et al. similarly suggested that upper zone mediastinal dissection is not needed for lower lobe tumors ≤1 cm [2].

Sub-analysis of the JCOG0802/WJOG4067L trial showed that 10.5% of patients with pure solid nodules had lymph node metastasis (5.4% mediastinal), whereas only 1.5% of part-solid GGN patients had nodal metastasis (0.5% mediastinal). Thus, mediastinal dissection may have minimal impact on staging in GGN cases, while selective dissection is advised in solid nodules [3,4].

**2. Subcarinal lymph node dissection**

Subcarinal node dissection is performed by more than half of institutions in cases of middle and lower lobe tumors. However, for upper lobe tumors, over half of the institutions responded that it is generally not performed, indicating that tumor location is a key determinant.

Past studies have supported omitting subcarinal dissection in upper lobe NSCLC [1,5,6]. Aokage et al. reviewed 1099 patients with upper lobe NSCLC and concluded that subcarinal node dissection could be omitted, particularly in clinical N0 squamous cell carcinoma [6]. However, Eckardt et al. observed a 1.6% subcarinal metastasis rate in 5577 NSCLC patients and advocated for routine dissection or sampling in all lobectomies, regardless of tumor location [7].

Therefore, whether subcarinal dissection can be omitted remains controversial.

**3. Pulmonary ligament lymph node dissection**

In lower lobe tumors, 71% of institutions reported performing pulmonary ligament (#9) node dissection in all cases, compared to only 8% for upper/middle lobe tumors.

Several studies have explored the necessity of #9 dissection. Yazgan et al. reported no significant impact on staging or survival in 840 upper lobectomy patients [8]. Maniwa et al. studied 352 cT1 lower lobe NSCLC patients and found 2.6% had #8/#9 node metastases. Notably, none occurred in S6 tumors, leading to the suggestion that #8/#9 dissection may be omitted in these cases [9].

These findings support tumor location as a key factor in determining the necessity of pulmonary ligament node dissection.

**4. En Bloc lymph node dissection**

Institutional opinions on the necessity of en bloc lymph node dissection vary widely:
• 14 institutions (22%) deemed it “required”
• 27 (43%) considered it “ideally meaningful but technically difficult”
• 18 (29%) answered “meaningless”
• 3 (5%) considered it “technically impossible”

Definitions also varied: about half (49%) defined it as “regional lymph node dissection without division.” Nagata et al. reported that en bloc dissection—defined as lobectomy with hilar and mediastinal lymph nodes resected together without separation—was associated with improved survival and was feasible, especially in patients with potential N-positive NSCLC [10]. However, no other studies have confirmed these findings.

**5. Lymphatic flow**

Responses to lymphatic flow-related questions were diverse. Although lymphatic drainage is generally considered to follow a caudal-to-cranial direction into the thoracic duct or right lymphatic duct and then into the subclavian veins [11], the actual flow is highly individualized and complex.

Most previous studies focus on sentinel node detection [12–14]. Kawakami et al. used CT lymphography with lipiodol injection to assess lymphatic pathways in lung cancer. They found that sentinel lymph nodes were outside the lobe-specific area in 13.9% of cases, with some located in tumor-free lobes [15]. Notably, two upper lobe cancers had sentinel nodes in #7 (subcarinal). These findings suggest that lymphatic flow is not strictly lobe-based and support the value of systematic lymph node dissection for accurate staging.

**Supplemental text S8.** Discussion of lymph node dissection and en bloc dissection in esophageal cancer.

**Lymph node dissection in esophagectomy**

The effectiveness of lymph node dissection (LND) in esophagectomy has been addressed in multiple studies. A landmark paper by Kato et al. reported 5-year survival rates of 43% and 61% for patients undergoing two-field and three-field dissections, respectively—a statistically significant difference [16].

While the overall evidence supports the oncologic benefit of LND in esophageal cancer, Tachimori et al. emphasized that the efficacy index (EI) of mediastinal LND differs based on tumor location. Nevertheless, they recommended upper mediastinal dissection across all thoracic squamous cell carcinomas [17].

Dissection of nodes around the recurrent laryngeal nerves—especially 106recR—is both oncologically valuable and surgically challenging due to its association with postoperative recurrent nerve palsy and aspiration pneumonia. Nonetheless, this station shows a high therapeutic impact, even in superficial tumors [18,19].

A prospective study by Xu et al. demonstrated that subcarinal lymph node dissection improved 5-year survival (44.2% vs. 30.0%) and that subcarinal metastasis correlated with lymph node size, invasion depth, and tumor location [20].

Regarding pulmonary ligament lymph nodes, Huang et al. reported a 10% metastasis rate in patients undergoing video-assisted thoracoscopic esophagectomy (VATS-E), suggesting that this region warrants routine dissection [21].

***International variation in practice***

A recent multicenter survey in the Netherlands explored the extent and anatomical definitions of lymphadenectomy during minimally invasive esophagectomy (MIE) [22]. The study revealed significant inter-institutional variability in both the routine extent of lymph node dissection and the anatomical boundaries of nodal stations. These variations may hinder outcome comparability and impede the development of standardized treatment strategies. The authors highlighted the value of such multi-institutional surveys for fostering consensus—particularly when involving different specialties in overlapping anatomical fields, as done in the present study.

**Understanding lymphatic flow in esophageal cancer**

The results of our questionnaire indicate no unified understanding of mediastinal lymphatic flow among esophageal and thoracic surgeons.

Cadaveric anatomical studies from Japan provide some insights. In the esophagus, the lymphatic channels located above the submucosal layer lack valves, permitting bidirectional (ascending and descending) lymph flow [23]. This contributes to the early and extensive spread of esophageal cancer, a key difference from lung cancer.

Murakami et al. reported that 43% of the thoracic esophagus drains directly into the thoracic duct [24]. Cadaveric evidence also suggests right-sided lymphatic drainage follows a longitudinal, multi-station path, whereas left-sided drainage is often limited by the aortic arch and leads directly to the thoracic duct. Additionally, cross-connections between the middle and lower mediastinum are frequent [25], and no consistent unidirectional flow has been confirmed.

**Biological lymphatic flow: functional evidence**

Radioisotope-based studies (e.g., 99mTc colloid injection) provide functional insight into lymphatic flow patterns. According to Tanabe et al. [26], the general directional tendencies are:

- Upper thoracic (Ut): Predominantly ascending flow
- Middle thoracic (Mt): Bidirectional (ascending and descending)
- Lower thoracic (Lt): Predominantly descending flow

Notably, the right upper mediastinum consistently showed high uptake, reaffirming the central role of station 106recR in esophageal cancer surgery.

In canine models, dye injection studies of the lower esophagus and gastric fundus revealed bidirectional lymphatic flow in the esophagus, but exclusively downward drainage from the gastric fundus—supporting the concept that gastric-to-mediastinal lymphatic spread is limited [26].

**Conclusion**

These findings underscore the complex, multi-directional nature of esophageal lymphatic drainage and the rationale for wide-field lymphadenectomy in esophagectomy. The lack of consistent anatomical or physiological definitions complicates the development of a unified surgical strategy, but collaborative, cross-disciplinary approaches like this study may help bridge these gaps.

**References**

1. Deng HY, Zhou J, Wang RL, Jiang R, Zhu DX, Tang XJ, et al. Lobe-specific lymph node dissection for clinical early-stage (cIA) peripheral non-small cell lung cancer patients: what and how? Ann Surg Oncol 2020;27(2):472–480. doi:10.1245/s10434-019-07926-3
2. Yang MZ, Hou X, Liang RB, Lai RC, Yang J, Li S, et al. The incidence and distribution of mediastinal lymph node metastasis and its impact on survival in patients with non-small-cell lung cancers 3 cm or less: data from 2292 cases. Eur J Cardiothorac Surg 2019;56(1):159–166. doi:10.1093/ejcts/ezy479
3. Saji H, Okada M, Tsuboi M, Kato H, Tada H, Mitsudomi T, et al. Segmentectomy versus lobectomy in small-sized peripheral non-small-cell lung cancer (JCOG0802/WJOG4607L): a multicentre, open-label, phase 3, randomised, controlled, non-inferiority trial. Lancet 2023;401(10373):1604–1615. doi:10.1016/S0140-6736(23)00568-5
4. Maniwa T, Okami J, Miyoshi T, Wakabayashi M, Yoshioka H, Mimae T, et al. Lymph node dissection in small peripheral lung cancer: Supplemental analysis of JCOG0802/WJOG4607L. J Thorac Cardiovasc Surg 2024;168(3):674–683.e1. doi:10.1016/j.jtcvs.2023.11.023
5. Wang RL, Zhang X, Chen F, Chen M, Zhang J. Subcarinal lymph node dissection in upper lobectomy for early-stage NSCLC: is it necessary? Mol Clin Oncol 2023;18(5):73. doi:10.3892/mco.2023.2659
6. Aokage K, Yoshida J, Ishii G, Hishida T, Nishimura M, Nagai K. Subcarinal lymph node metastasis in patients with resected stage I non-small-cell lung cancer. Lung Cancer 2010;70(2):175–180. doi:10.1016/j.lungcan.2010.01.009
7. Eckardt J, Licht PB. Is complete mediastinal lymphadenectomy required in routine surgery for NSCLC? Ann Thorac Surg 2017;103(2):466–472. doi:10.1016/j.athoracsur.2016.07.038
8. Yazgan S, Duman E, Onur S, Yalcin A, Demir A. Does No. 9 lymph node dissection improve survival in upper lobe non-small cell lung cancer? Acta Chir Belg 2023;123(6):412–418. doi:10.1080/00015458.2023.2240452
9. Maniwa T, Ohue M, Kanzaki R, Shintani Y, Okami J. Metastatic pathways to the lower zone by segment in patients with clinical T1 lower lobe non-small cell lung cancer. Gen Thorac Cardiovasc Surg 2024;72(10):684–689. doi:10.1007/s11748-024-02021-1
10. Nagata T, Aoki M, Maeda K, Kamimura G, Takeda A, Sato M, et al. En bloc resection of a primary tumor and lymph nodes in non-small-cell lung cancer. Ann Thorac Cardiovasc Surg 2024;30(1):24-00108. doi:10.5761/atcs.oa.24-00108
11. Bujoreanu I, Gupta V. Anatomy, Lymph Nodes. In: StatPearls [Internet]. Treasure Island (FL): StatPearls Publishing; 2024. PMID:32491649
12. Imai K, Minamiya Y, Saito H, Nakagawa T, Ito M, Ono T, et al. Detection of pleural lymph flow using indocyanine green fluorescence imaging in non-small cell lung cancer surgery: a preliminary study. Surg Today 2013;43(3):249–254. doi:10.1007/s00595-012-0237-2
13. Minamiya Y, Nakagawa T, Saito H, Ito M, Ono T, Motoyama S, et al. Thoracoscopic detection of sentinel lymph nodes in patients with non-small-cell lung cancer. Eur J Cardiothorac Surg 2007;32(5):753–757. doi:10.1016/j.ejcts.2007.07.005
14. Takeda-Harada AH, Watanabe Y, Nagata T, Aoki M, Umehara T, Suzuki S, et al. Detection of alternative subpleural lymph flow pathways using indocyanine green fluorescence. Surg Today 2018;48(6):640–648. doi:10.1007/s00595-018-1631-1
15. Kawakami Y, Takizawa H, Toba H, Kawakita N, Yoshida M, Kondo K, et al. Diversity of lymphatic flow in patients with lung cancer revealed by computed tomography lymphography. Interact Cardiovasc Thorac Surg 2021;33(6):871–878. doi:10.1093/icvts/ivab204
16. Kato H. Lymph node dissection for thoracic esophageal carcinoma. Two- and 3-field lymph node dissection. Ann Chir Gynaecol 1995;84(2):193–199.
17. Tachimori Y, Ozawa S, Numasaki H, et al. Efficacy index of lymph node dissection in thoracic esophageal cancer. Esophagus 2016;13(1):1–7. doi:10.1007/s10388-015-0494-5
18. Chen X, Yu J, Lu M, Wu J, Xu Z, Zhang X, et al. Clinical significance of lymph node dissection along the recurrent laryngeal nerves during esophagectomy. J Gastrointest Oncol 2021;12(4):1223–1227. doi:10.21037/jgo-21-146
19. Mizutani M, Takeuchi H, Fukuda K, et al. Distribution of lymphatic vessels and nerves around the recurrent laryngeal nerve in the thoracic inlet. Surg Radiol Anat 2006;28(4):333–338. doi:10.1007/s00276-006-0102-z
20. Xu B, Chen H, Zhang Q, Chen P, Liu Q, Chen M. Value of subcarinal lymph node dissection in esophageal cancer surgery: a case-control study. Medicine (Baltimore) 2022;101(43):e31593. doi:10.1097/MD.0000000000031593
21. Huang S, Xu L, Luo J, Zhang M, Wang X, Zeng Y. Necessity of inferior pulmonary ligament lymph node dissection in thoracoscopic esophagectomy. Medicine (Baltimore) 2021;100(23):e26302. doi:10.1097/MD.0000000000026302
22. Ketel MHM, Ruurda JP, van der Sluis PC, Dikken JL, Nieuwenhuijzen GAP, van Hillegersberg R. Extent of lymphadenectomy and anatomical definitions of lymph node stations during minimally invasive esophagectomy: results of a national survey. Ann Surg Oncol 2024;31(1):e36–e44. doi:10.1245/s10434-023-14062-2
23. Wang Y, Zhu L, Xia W, Wang F. Lymphatic vessel structure and function in esophageal cancer. Cancer Manag Res 2018;10:6295–6303. doi:10.2147/CMAR.S185585
24. Murakami G, Tsurumoto T, Hata T, Taguchi T, Mukaiya M, Kimura W, et al. Anatomical study of lymphatic drainage of the esophagus in humans. Surg Radiol Anat 1994;16(4):399–407. doi:10.1007/BF01627803
25. Saito H, Sato T, Miyazaki M. Morphological and topographical study of lymphatic vessels of the human thoracic esophagus. Surg Radiol Anat 2007;29(7):531–542. doi:10.1007/s00276-007-0253-9
26. Tanabe G, Inoue T, Oka Y, Sano Y, Ishibashi Y, Kusaka M. Study on lymphatic drainage of the esophagus and the stomach using 99mTc-Rhenium colloid and dye injection methods. Nihon Geka Gakkai Zasshi 1986;87(3):315–323. (in Japanese)

**Supplemental Table S3.** Search expression for making of statement.

| **The part of lymph node dissection** | **Search formula for PubMed Search** |
| --- | --- |
| **About left recurrent nerve lymph node dissection** | ("esophageal cancer" OR "esophageal carcinoma" OR “lung cancer”) AND ("left recurrent" OR "left laryngeal") AND ("lymph node dissection" OR "lymphadenectomy") |
| **About carinal lymph node dissection** | ("esophageal cancer" OR "esophageal carcinoma" OR “lung cancer”) AND ("tracheal bifurcation" OR "carina" OR "subcarinal") AND ("lymph node dissection" OR "lymphadenectomy") |
| **About pulmonary ligament lymph node dissection** | ("esophageal cancer" OR "esophageal carcinoma" OR “lung cancer”) AND ("pulmonary ligament" OR "inferior mesenteric" OR "lower mediastinal") AND ("lymph node dissection" OR "lymphadenectomy") |

**Supplemental Figure S1.** Summary of study methods.

Assembling the Steering, Expert and Research Committees in each division

Development of Clinical Questions (in collaboration with the lung and esophageal divisions)

Design of Questionnaire and Literature Search

Formulation of Statements and Voting via Delphi Method

Presentation at the 37^th^ Annual Meeting of the Japan Society for Endoscopic Surgery

Questionnaire Tabulation

**
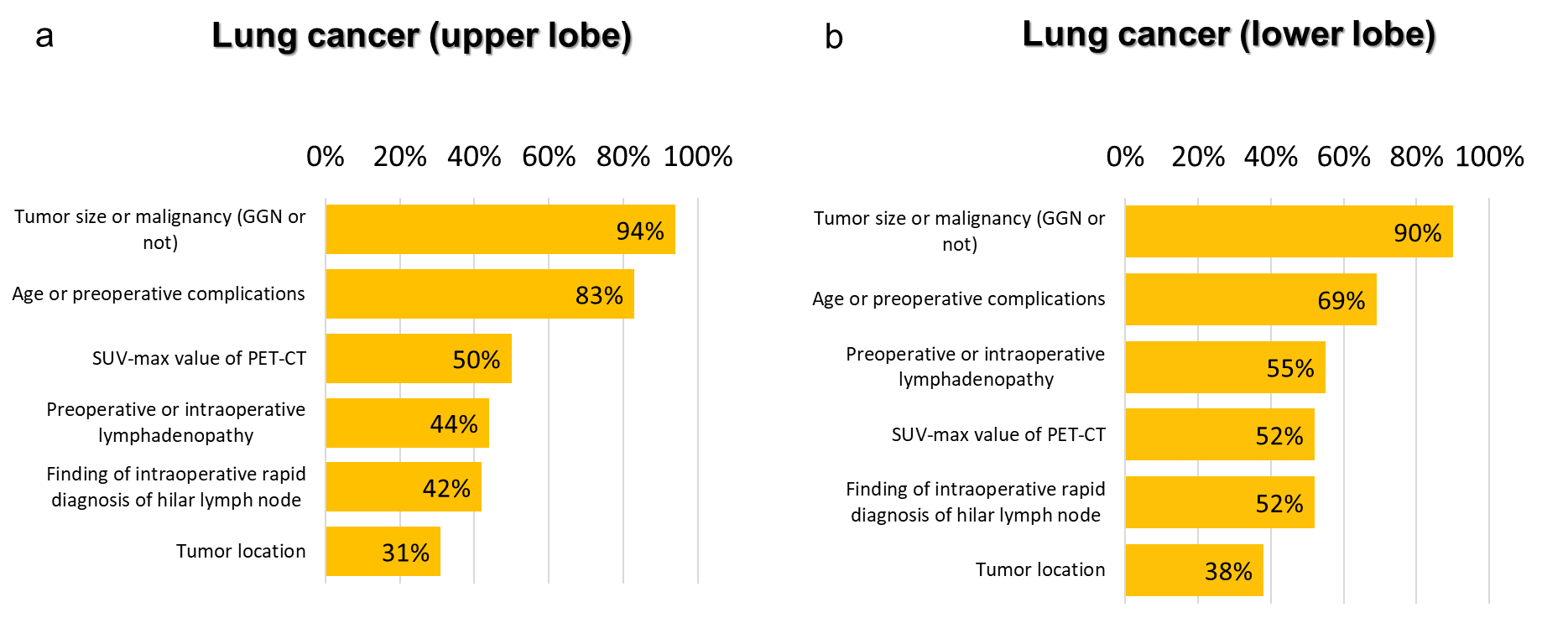
Supplemental Figure S2.** Criteria for left recurrent nerve lymph node dissection in lung cancer: (a) left upper lobe, (b) left lower lobe.

**Supplemental Figure S3.** Criteria for left recurrent nerve lymph node dissection in esophageal cancer.

**
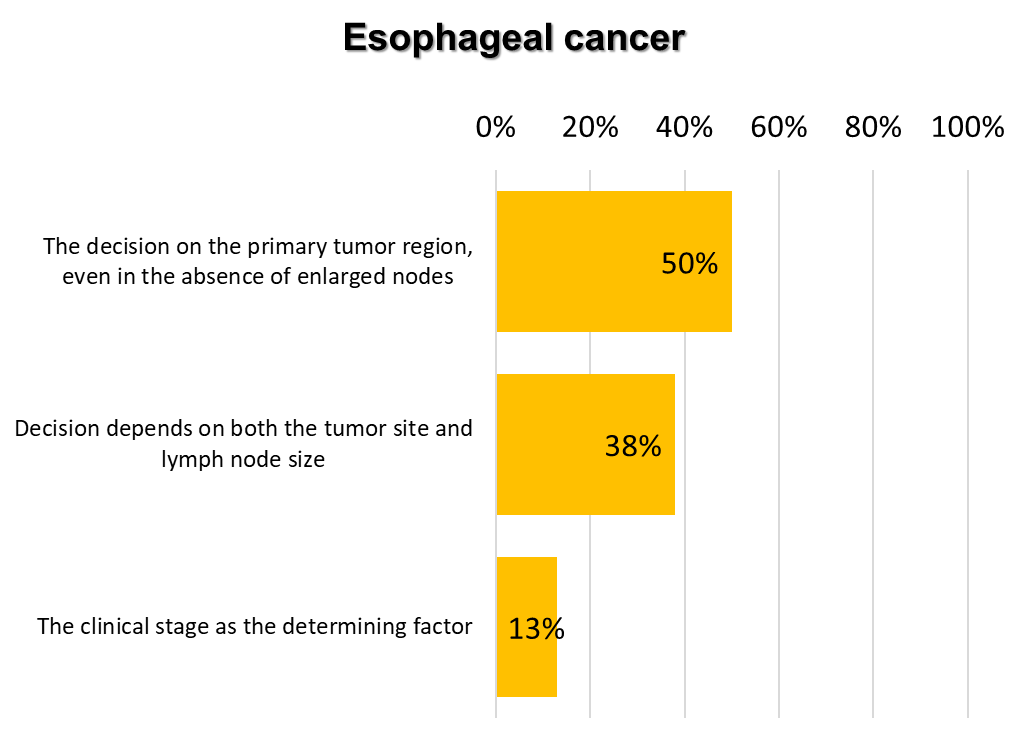
**

**Supplemental Figure S4.** Criteria for subcarinal lymph node dissection in lung cancer (lower lobe).

**
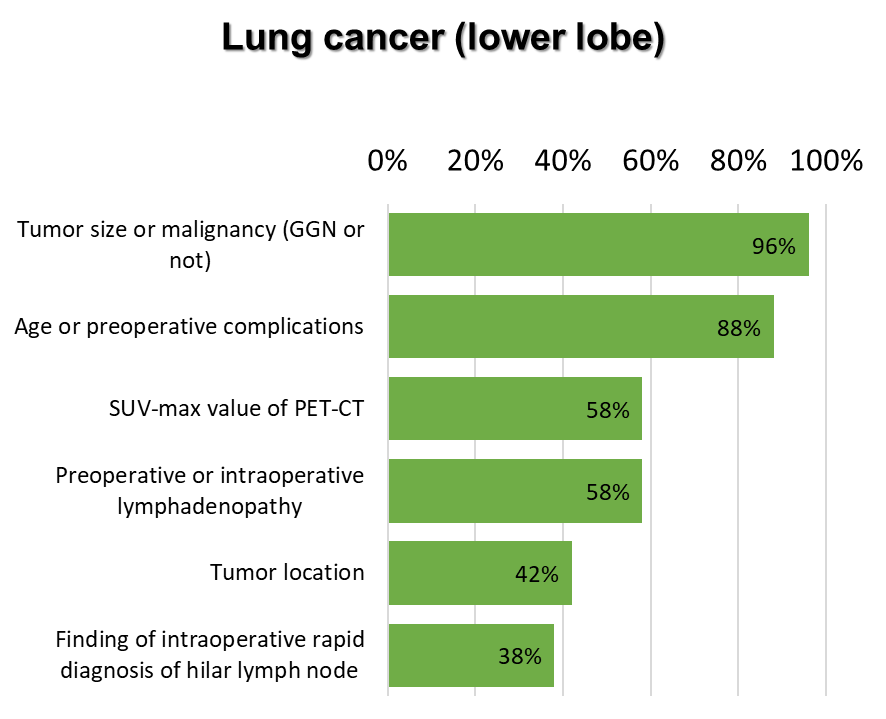
**

**Supplemental Figure S5.** Responses to “Are subcarinal lymph node dissections different between left and right sides?” (lung cancer)

**
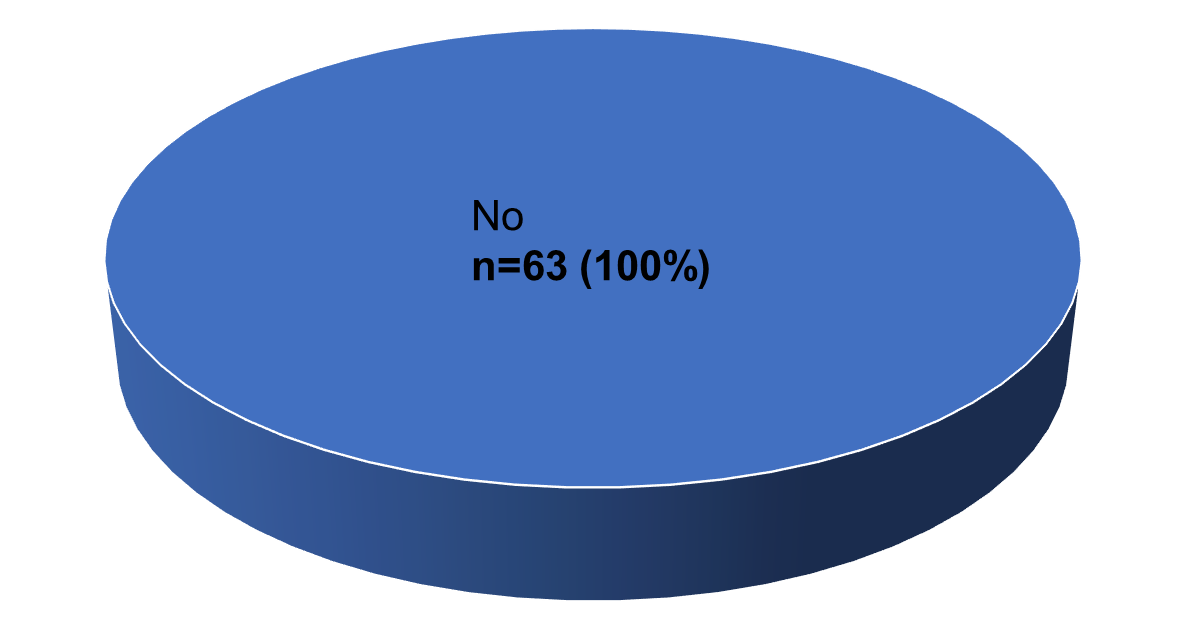
**

**Supplemental Figure S6.** Criteria for subcarinal lymph node dissection in esophageal cancer.


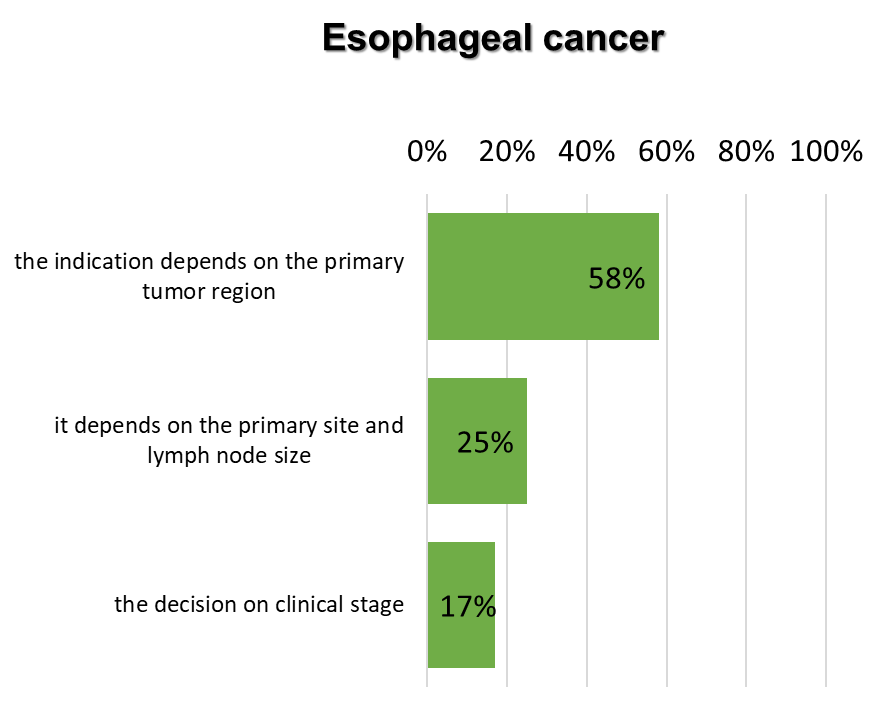


**Supplemental Figure S7.** Expectations for subcarinal lymph node dissection: (a) lung cancer, (b) esophageal cancer.

**
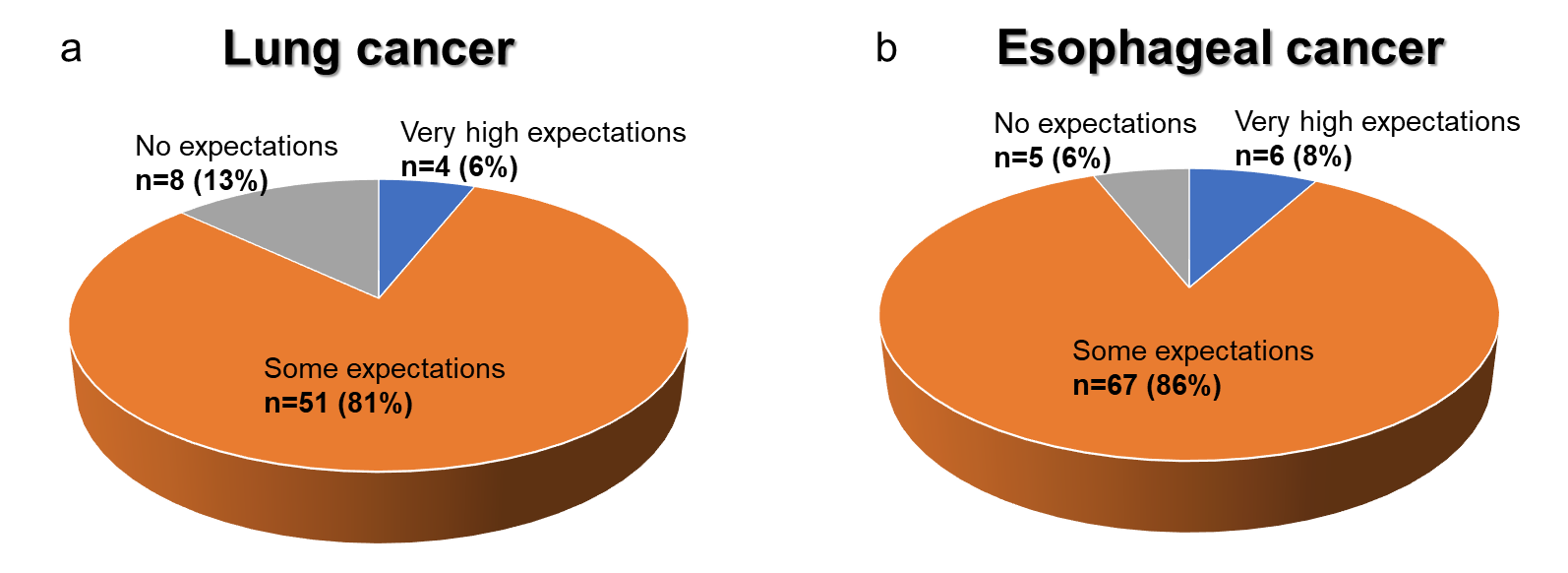
**

**Supplemental Figure S8.** Upstream lymphatic flow of subcarinal lymph nodes: (a) lung cancer, (b) esophageal cancer.

**
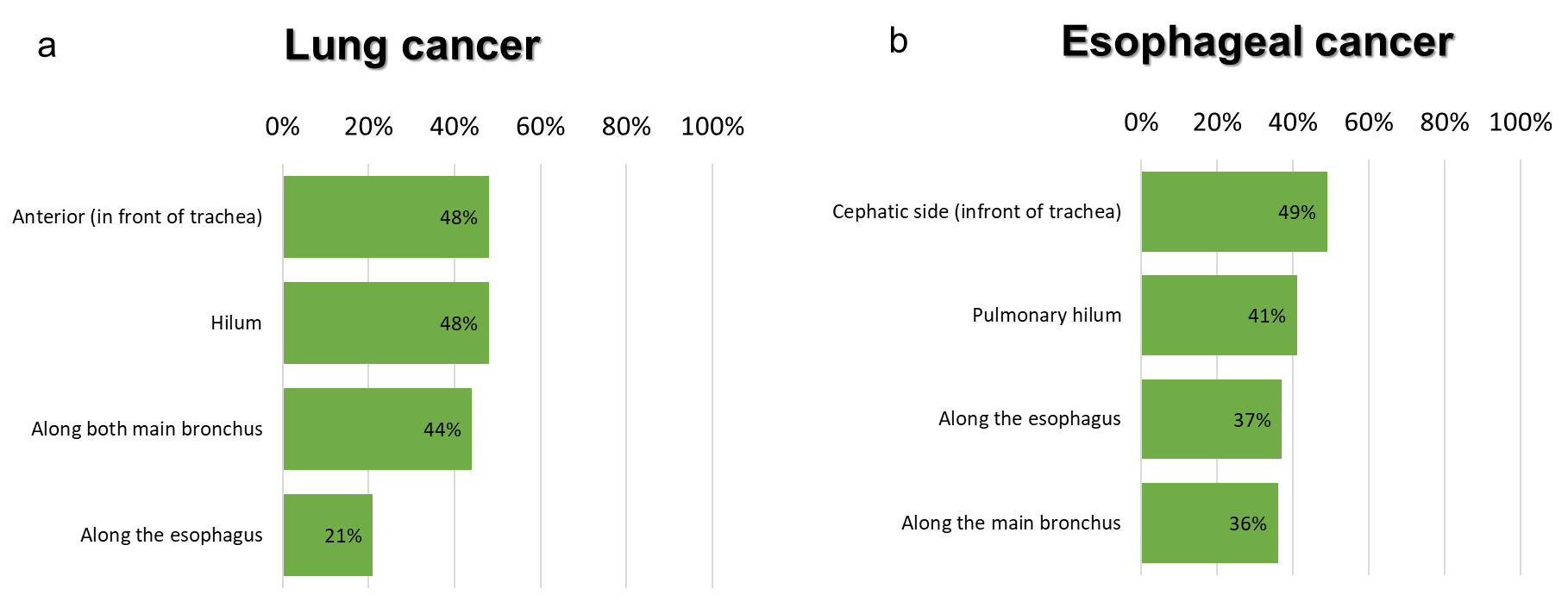
**

**Supplemental Figure S9.** Downstream lymphatic flow of subcarinal lymph nodes: (a) lung cancer, (b) esophageal cancer.

**
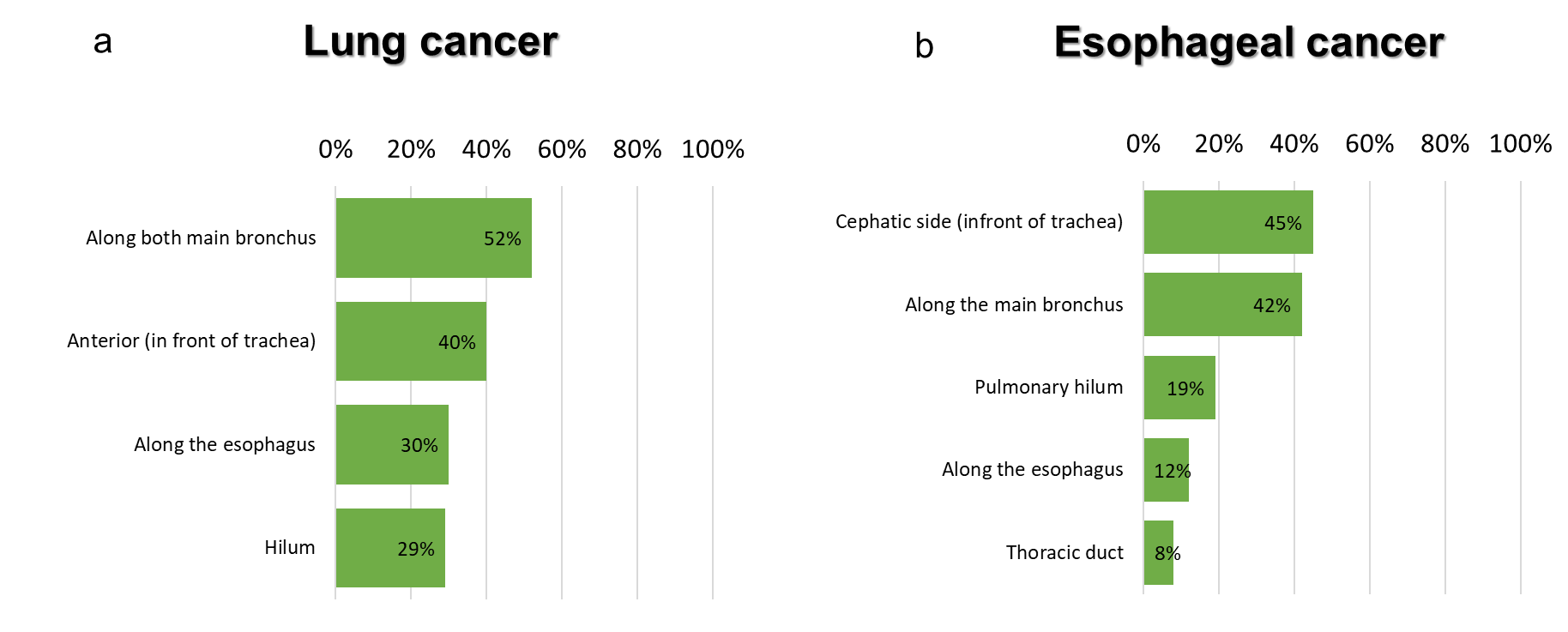
**

**Supplemental Figure S10.** Criteria for pulmonary ligament lymph node dissection in lung cancer.

**
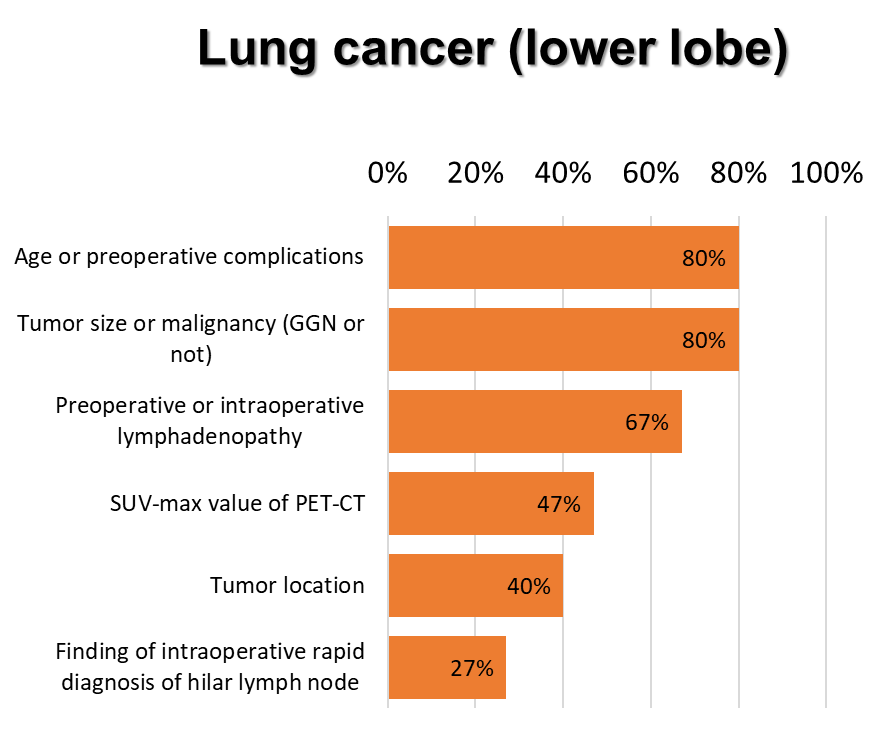
**

**Supplemental Figure S11.** Responses to “Are subcarinal lymph node dissections different between left and right sides?” (lung cancer).

**
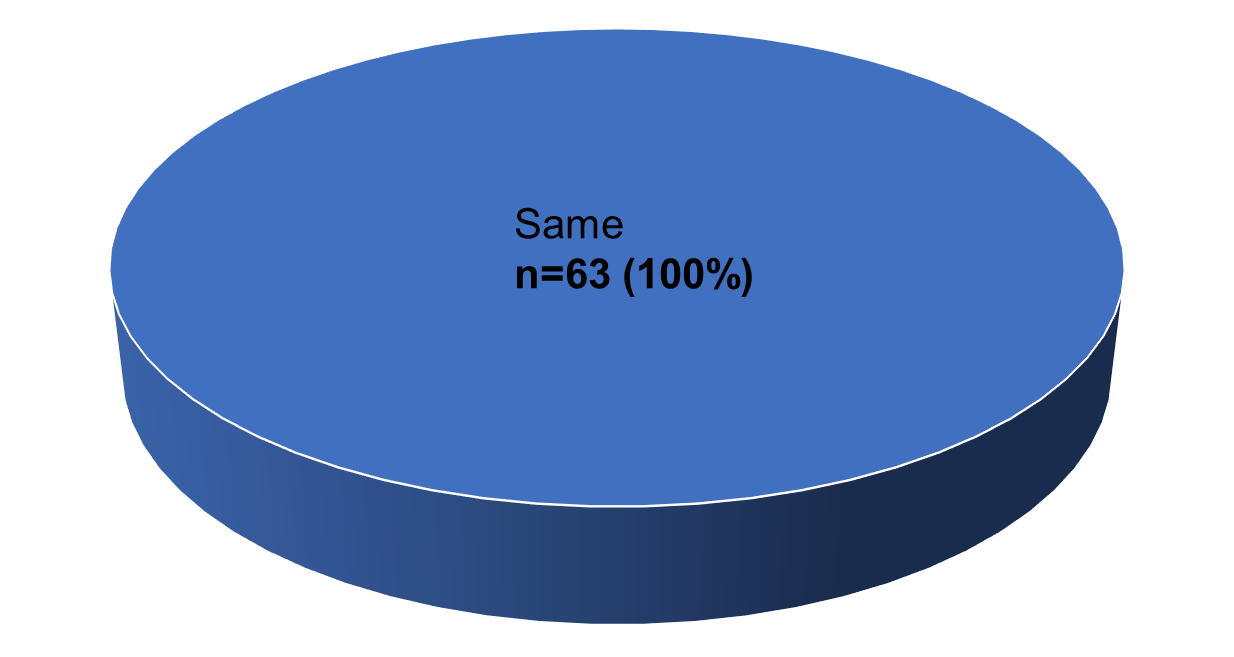
**

**Supplemental Figure S12.** Criteria for pulmonary ligament lymph node dissection in esophageal cancer.

**
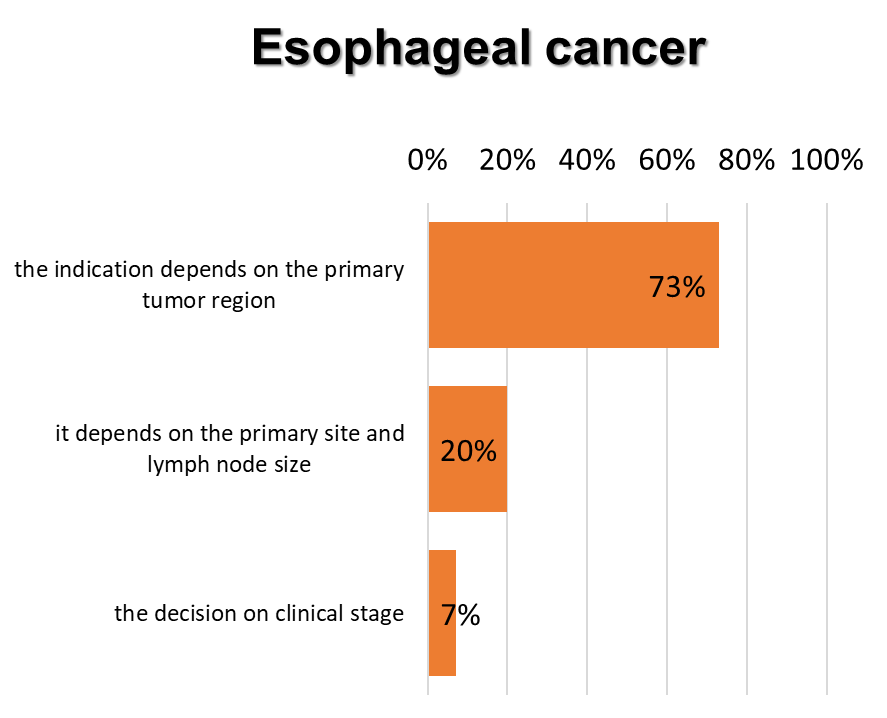
**

**Supplemental Figure S13.** Expectations for pulmonary ligament lymph node dissection: (a) lung cancer, (b) esophageal cancer.

**
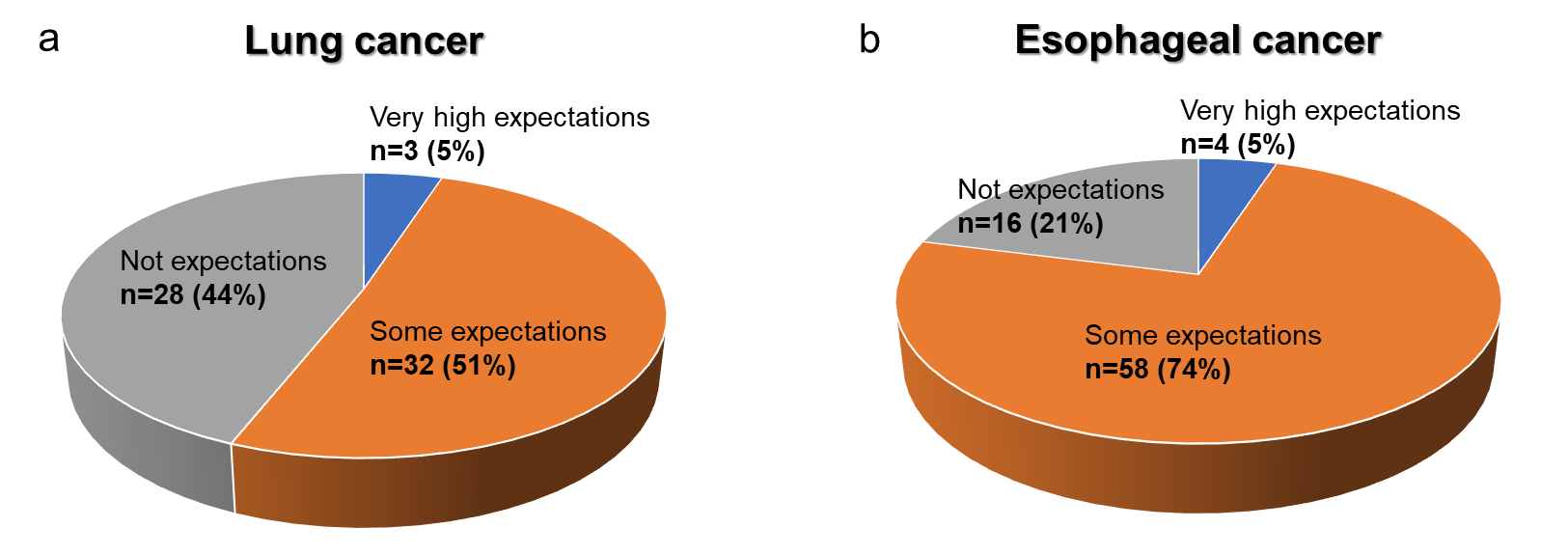
**

**Supplemental Figure S14.** Upstream lymphatic flow of pulmonary ligament lymph nodes: (a) lung cancer, (b) esophageal cancer.

**
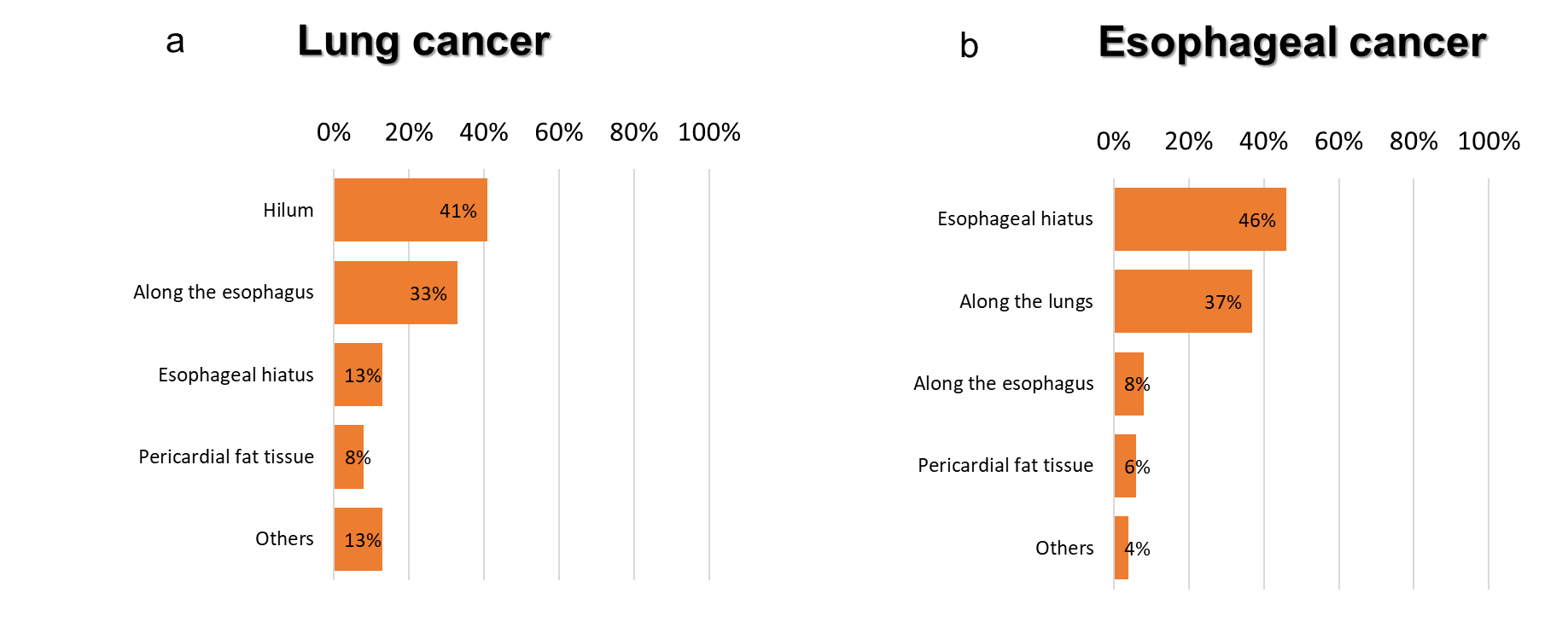
**

**Supplemental Figure S15.** Downstream lymphatic flow of pulmonary ligament lymph nodes: (a) lung cancer, (b) esophageal cancer.


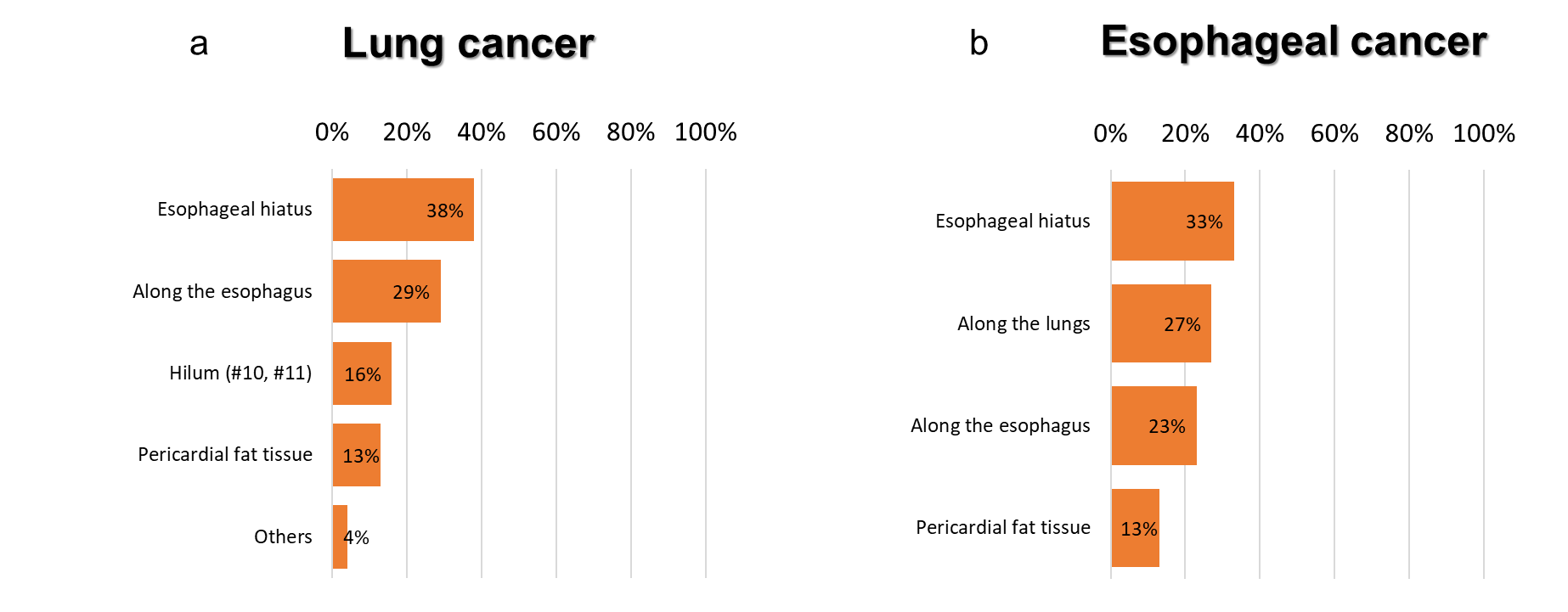


**Supplemental Figure S16.** Responses to “Is en bloc lymph node dissection required?”: (a) lung cancer, (b) esophageal cancer.

**
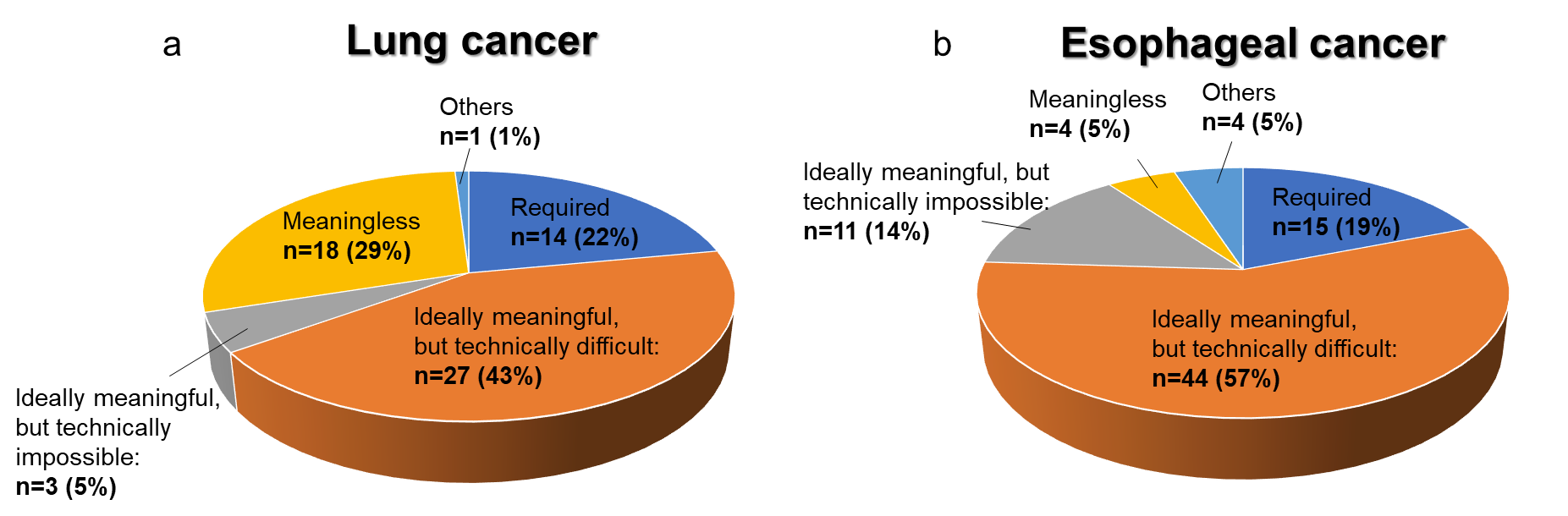
**

**Supplemental Figure S17.** T Definitions of “en bloc” lymph node dissection: (a) lung cancer, (b) esophageal cancer.

**
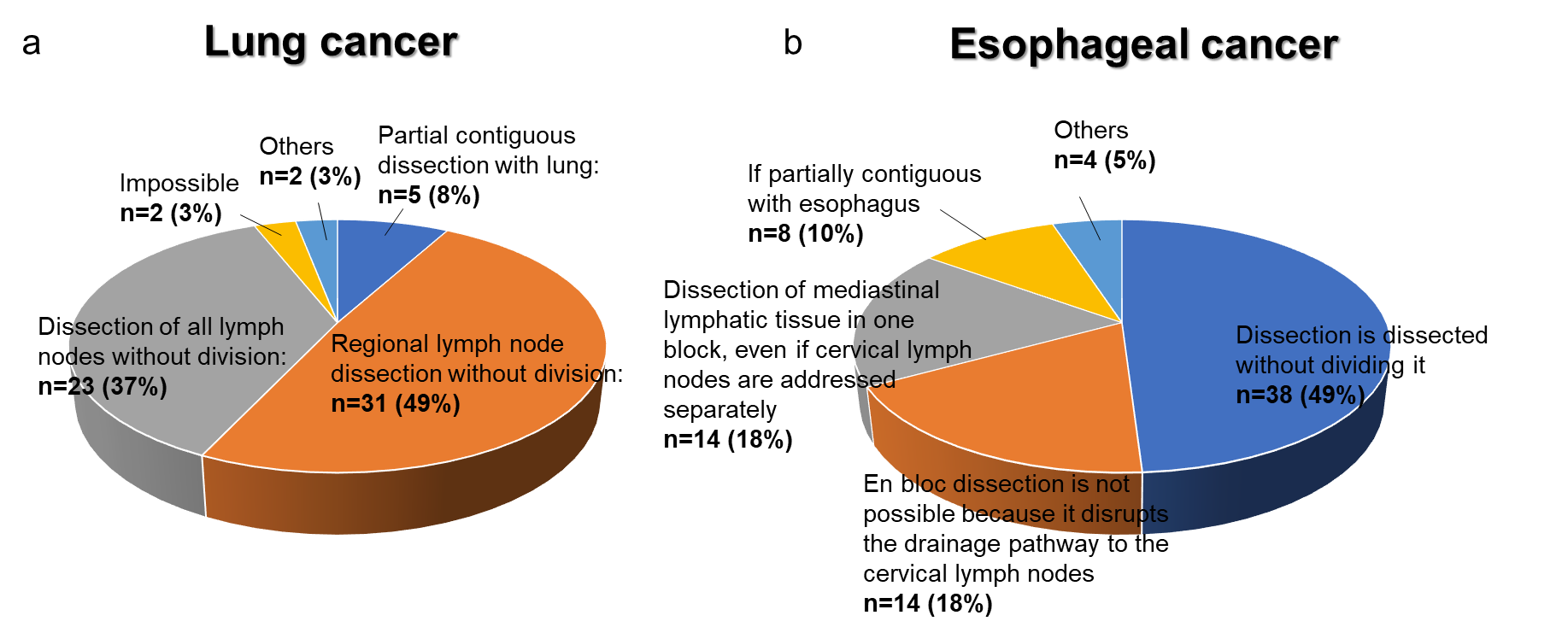
**
